# Activation of mammalian target of rapamycin mediates rat pain-related responses induced by BmK I, a sodium channel-specific modulator

**DOI:** 10.1186/1744-8069-9-50

**Published:** 2013-10-08

**Authors:** Feng Jiang, Xue-Yan Pang, Qing-Shan Niu, Li-Ming Hua, Ming Cheng, Yong-Hua Ji

**Affiliations:** 1 Lab of Neuropharmacology & Neurotoxicology, Shanghai University, 200444 Shanghai, P.R. China; 2 Brudnick Neuropsychiatric Research Institute, and Program In Neuroscience, University of Massachusetts Medical School, 01604, Worcester, MA, USA

**Keywords:** BmK I, mTOR, p70S6K, 4E-BP1, Rapamycin, Pain, Mirror-image mechanical hypersensitivity

## Abstract

The mammalian target of rapamycin (mTOR) is known to regulate cell proliferation and growth by controlling protein translation. Recently, it has been shown that mTOR signaling pathway is involved in long-term synaptic plasticity. However, the role of mTOR under different pain conditions is less clear. In this study, the spatiotemporal activation of mTOR that contributes to pain-related behaviors was investigated using a novel animal inflammatory pain model induced by BmK I, a sodium channel-specific modulator purified from scorpion venom. In this study, intraplantar injections of BmK I were found to induce the activation of mTOR, p70 ribosomal S6 protein kinase (p70 S6K) and eukaryotic initiation factor 4E-binding protein 1 (4E-BP1) in rat L5-L6 spinal neurons. In the spinal cord, mTOR, p70 S6K and 4E-BP1 were observed to be activated in the ipsilateral and contralateral regions, peaking at 1–2 h and recovery at 24 h post-intraplantar (i.pl.) BmK I administration. In addition, intrathecal (i.t.) injection of rapamycin – a specific inhibitor of mTOR – was observed to result in the reduction of spontaneous pain responses and the attenuation of unilateral thermal and bilateral mechanical hypersensitivity elicited by BmK I. Thus, these results indicate that the mTOR signaling pathway is mobilized in the induction and maintenance of pain-activated hypersensitivity.

## Introduction

Scorpion envenomation is known to release neurotoxins that induce long term pain sensation, edema and hypersensitivity [1-3]. Various neurotoxic polypeptides have been purified from the venom of the scorpion *Buthus martensi* Karsch (BmK) and have been shown to be specific modulators of the voltage gated sodium channel (VGSC) [4-9]. Among them, BmK I (receptor site 3 VGSC-specific modulator) has been demonstrated to be the primary contributor of envenomation-associated pain [10,11] as a result of its ability to increase persistent currents of tetrodotoxin-resistant (TTX-R) sodium channels and by delaying the inactivation phase of VGSCs [10]. As a novel pain model, many clinical pain symptoms, such as persistent spontaneous responses, thermal hypersensitivity and bilateral (mirror-image) mechanical hypersensitivity, could be well mimicked by intraplantarly (i.pl.) injection of BmK I in rat left hind paw. Interestingly, rat injected with BmK I always rolls on the floor suddenly, strongly shakes and bites the injected paw, and sometimes accompanied by shriek for several seconds. The unique and extreme spontaneous nociceptive behavior was defined as paroxysmal pain-like behavior [10]. Significant changes in spinal neuronal activities [12,13] were observed to be associated with BmK I-induced pain. However, it is still unclear how downstream intracellular activities in the central nervous system (CNS) amplify responses to BmK I-induced nociception.

The mammalian target of rapamycin (mTOR), a serine/threonine protein kinase, has been regarded as the hub of the intracellular signaling network as various types of signals converge on mTOR, thereby controlling the intracellular processes governing homeostasis [14,15]. Activated mTOR can then phosphorylate downstream molecules such as p70 ribosomal S6 protein kinase (p70 S6K) on two residues [16,17] which can then activate S6 ribosomal proteins to initiate new protein synthesis [18]. In addition, another downstream molecule, known as eukaryotic initiation factor 4E-binding protein 1 (4E-BP1), can be phosphorylated by mTOR on at least four sites [19,20]. Upon phosphorylation 4E-BP1 (p-4E-BP1) permits the release of eukaryotic translation initiation factor 4E (eIF4E) to associate with eiF-4G, thereby facilitating the loading of the ribosome onto the mRNA [21].

The mTOR-dependent mRNA translation pathway, which can be specifically inhibited by rapamycin [22,23], is one of the most important mechanisms that regulates neuronal excitability and modulates long-term plasticity associated with learning and memory [24-26]. Since synaptic plasticity is responsible for central sensitization induced by noxious stimuli in the CNS [27,28], it may suggest that the mTOR pathway is essential to chronic pain. Although it remains less clear if low levels of activated mTOR is sufficient to mediate acute pain in normal conditions [29], increasingly studies reveal that mTOR-dependent signaling cascades are involved in pain hypersensitivity [30,31].

However, the current knowledge regarding the linkage between the mTOR-dependent pathway and pain-related behaviors attributed to scorpion envenomation requires further research. In this study, the following questions of interest were investigated: 1) does the level of activated mTOR and its respective downstream molecules change under BmK I-induced pain state? 2) What is the distribution of p-mTOR and its downstream molecules? 3) Does the inhibition of spinal mTOR activity attenuate BmK I-induced pain? and if so, 4) in which phase does the mTOR-dependent pathway take effect?

## Materials and Methods

### Experimental animals

Adult male Sprague–Dawley rats (220 to 250 g; Shanghai Experimental Animal Center, Chinese Academy of Sciences) were housed under a 12-h light–dark cycle at 21–23°C with 50% humidity. Each cage contains five rats with water and food available ad libitum. The rats were habituated to the laboratory 5 days before experiments. All procedures and testing occurred during the light cycle. European Community guidelines for the use of experimental animals and guidelines of International Association for the Study of Pain (IASP) for pain research in conscious animals were applied [32].

### BmK I preparation and administration

The crude BmK venom collected by electrical stimulation was purchased from an individual scorpion culture farm in Henan Province, China. BmK I was purified according to our previously described protocol [33]. The purity of BmK I used in this study was assessed to be a single peak on mass spectrum. BmK I was dissolved in physiological saline (0.9% NaCl). 50 μl diluted BmK I solution (0.2 μg/μl) and i.pl. injected into the left hind paw of the animal.

### Drugs

Rapamycin (TOCRIS bioscience, MO), the specific inhibitor of mTOR, was dissolved in saline/DMSO mix comprising 25% v/v DMSO [30]. CCI-779 (Selleck Chemicals LLC, TX) was prepared in pure ethanol as a stock solution on the day of the experiment and diluted to 250 μM in 0.15 M NaCl, 5% polyethylene glycol 400, 5% Tween 20 [34]. A volume of 10 μl of drug solution, or equivolume vehicle, was injected intrathecally (i.t.) by direct lumbar puncture between the L5 and L6 in rats 30 min before or 2 h after the i.pl. administration of BmK I (n=8 for each group) as the previous description [35]. Briefly, rats were slightly anesthetized with ether. The i.t. injection was made with a 27-gauge, 1-inch sterile disposable needle connected to a 25 μl Hamilton syringe. Puncture of the dura was indicated by a reflexive flick of tail or formation of an "S" by the tail.

### Behavioral testing

#### *Measurement of spontaneous response behaviors*

A 20 × 20 × 30 cm^3^ transparent plexiglas box with a glass floor was fixed on a steel frame of 75 cm high above the experimental table covered with a mirror. Rats were placed in the boxes for habituation at least 30 min before the tests. After i.t. injection of rapamycin or vehicle, rats were allowed to recover from anesthesia for a short period of time. Subsequently, BmK I was subcutaneously injected in the left hind paw of rats at 30 min post-rapamycin treatment. The behaviors are determined by counting the number of flinches and the time duration spent in lifting and licking the injected hind paw during a 5 min interval for 2 h [36]. Following the observation of spontaneous behaviors exhibited by the rats, each experimental group (n=8) was examined for the sensitivity to mechanical or radiated heat stimuli.

#### *Measurement of paw withdrawal mechanical threshold (PWMT)*

Rats were placed in a plexiglas test box (20 × 20 × 30 cm) on a mesh floor (1 cm^2^ quadrate openings) for habituation 30 min before examination. Mechanical sensitivity was assayed by using a series of 10 calibrated von Frey filaments with forces from 0.6 to 26 g (58011, Stoelting Co., IL). Filaments were applied from underneath the metal mesh floor to the bilateral hind paws. Each filament was probed for the same duration of 2–3 s with an inter-stimulus interval of 10 s. The positive response is indicated by brisk withdrawal or flinching of the tested paw. The rat PWMT was defined as the lowest force that caused at least five withdrawals out of the ten consecutive applications [36]. One day before experiment, the rats’ PWMTs were measured as the baseline value.

#### *Measurement of paw withdrawal thermal latency (PWTL)*

Rat paw withdrawal thermal latency (PWTL) to radiant heat stimuli was determined as previously described [37]. Briefly, rats were placed on the surface of a 2 mm thick glass plate covered with the transparent plexiglas test box (20 × 20 × 30 cm^3^) for habituation at least 30 min before testing. Heat stimuli were provided with radiant heat stimulator (RTY-3, Xi’an Fenglan instrumental factory, China). The heat source was a high intensity projector halogen lamp bulb (150 W, 24 V) positioned under the glass floor 2 cm directly beneath the targeting area of hind paw. The diameter of the light spot on the floor surface was approximately 3 mm and an exposure time of 20 s was set to limit tissue injury. For each rat, five stimuli were administered and the stimuli interval was 10 min. Rat PWTL was determined by averaging the three middle values. One day before the experiment, the rat PWTLs were collected as baseline values.

Moreover, those who were responsible for data collection were blind to the treatment.

### Immunohistochemistry

Our previous study showed that the expression of c-fos, a marker recognized as neuronal excitability, reached the peak in rat spinal cord dorsal horn 2 h after BmK I administration [13]. Consequently, we chose the time point of 2 h to detect whether mTOR cascades were activated through immunohistochemistry assay.

Rats were anesthetized at time point of 2 h with pentobarbital sodium (60 mg/kg body weight) and perfused intracardially with 200 ml of sterile saline, followed by 400 ml of fixative containing 4% paraformaldehyde in 0.1 M phosphate buffer (PB; pH 7.4). The lumbar spinal cord was dissected and post-fixed in the same fixative for 12 hours at 4°C and then cryoprotected in 0.1 M PB containing 30% sucrose until the tissue sank to the bottom of the container. Frozen serial coronal sections (14 μm in thickness) were cut with a Microm cryostat (HM 525) and mounted on gelatin-coated glass slides. Nonspecific binding was blocked by incubation with 5% normal horse serum (S2000; Vector Laboratories, CA) in PBS with 0.3% Triton X-100.

For double labeling of p-mTOR/p-p70 S6K/p-4E-BP1 with NeuN/GFAP/Iba-1, L5 spinal cord sections were incubated for 48 h at 4°C with a mixture of rabbit monoclonal anti-p-mTOR (Ser2448 49F9, 1:200; #2976; Cell Signaling Technology, MA [38])/rabbit polyclonal anti-p-p70 S6K (Thr421/Ser424, 1:50; sc-7984-R; Santa Cruz, CA)/rabbit monoclonal anti-p-4E-BP1 (Thr36, 1:50; BS5047; Bioworld Technology, MN) and mouse monoclonal anti-NeuN (clone A60, 1:600; MAB377; Millipore Bioscience Research Reagents [31])/anti-GFAP (GA5, 1:200; #3670; Cell Signaling Technology, MA)/goat polyclonal anti-Iba-1 (1:50; ab5076; abcam, MA [39]). After rinsing with 0.01 M PBS for 15 min, the sections were then incubated with a mixture of donkey anti-rabbit IgG conjugated with Cy3 (1:500; 711-165-152; Jackson ImmunoResearch, PA) and anti-mouse IgG conjugated with FITC (1:200; 715-095-020; Jackson ImmunoResearch, PA)/anti-goat IgG conjugated with FITC (1:200; 705-095-003; Jackson ImmunoResearch, PA) for 6 h at room temperature. Control experiments were performed in parallel. For detection of p-mTOR/p-p70 S6K/p-4E-BP1, the sections were incubated with a one of the previously mentioned antibodies at 4°C for 48 h and then washed in 0.01 M PBS for 15 min. Subsequently sections were then incubated with their respective secondary antibodies conjugated with Cy3 or FITC. After the sections were rinsed in 0.01 M PBS, cover slips were applied. In order to exclude the potential false-positive results arising from antibody selection, the immunostaining results of p-mTOR, p-4E-BP1 and p-p70 S6K were independently validated by other different antibodies: anti-p-mTOR (Ser2448) antibody (#2971, Cell Signaling Technology), anti-p-4E-BP1 (Thr37/46) Rabbit mAb (#2855, Cell Signaling Technology) and anti-p-p70 S6 Kinase (Thr421/Ser424) antibody (#9204, Cell Signaling Technology). Related results could be found in supplementary material (Additional file 1: Figure S2, Additional file 2: Figure S3, Additional file 3: Figure S4).

All immunofluorescence-labeled sections were viewed and captured with an Olympus FV-1000 confocal system using sequential scanning to avoid fluorescence bleed-through. Double-labeled images were quantitatively evaluated with image analysis software (Image-Pro Plus 6.0). Area frequency of co-labeled cells in mTOR cascades positive cells was analyzed. The threshold of positive signals against noise signal or background was defined as shown in supplementary figure (Additional file 4: Figure S1 A-D). Intensities exceeding the threshold were accepted as true immunostaining. Three homolographic fields captured from lamina III-V were measured (Additional file 4: Figure S1 E). The average area in the three defined fields presents the immunoreaction level. Three rats were used for each group.

### Protein extraction and immunoblotting

Rats were anesthetized with intraperitoneal injection of sodium pentobarbital (60 mg/kg, i.pl.) and decapitated at various time points after the BmK I administration. The spinal cord of each rat was removed by pressure expulsion with saline into an ice-cooled glass dish where the lumbosacral enlargement was identified. Next, the section was labeled and snap-frozen in liquid nitrogen until further treatment. The sectioned tissues were homogenized at 4°C in radioimmune precipitation buffer [150 mM NaCl, 100 mM Tris (pH 8.0), 1% Triton X-100, 1% deoxycholic acid, 0.1% SDS, 5 mM EDTA and 10 mM NaF] supplemented with 1 mM sodium vanadate, 2 mM leupeptin, 2 mM aprotinin, 1 mM phenylmethylsulfonyl fluoride (PMSF), 1 mM DTT, and 2 mM pepstatin A. After centrifugation at 14,000 rpm for 15 min, the supernatant which contained the total cellular protein extract was collected and stored at -70°C. The protein concentration was determined using the Brandford assay (Sigma, MO).

Western blotting experiments were performed in order to confirm the temporal phosphorylation level of p-mTOR/p-p70 S6K/p-4E-BP1 following BmK I administration. Briefly, thirty micrograms of protein extracts were separated by SDS-polyacrylamide gel electrophoresis (SDS-PAGE), and transferred to a PVDF membrane. Nonspecific binding was blocked by incubation in 5% nonfat milk overnight at 4°C. The membranes were incubated with the following primary antibodies which were diluted in PBS-T containing 5% nonfat milk for 5 h at room temperature: rabbit polyclonal antibody against p-mTOR (Ser2448, 1:1000; #2971; Cell Signaling Technology, MA [31])/rabbit monoclonal antibody against mTOR (7C10, 1:1200; #2983; Cell Signaling Technology, MA)/rabbit polyclonal antibody against p-p70 S6K (Thr421/Ser424, 1:1500; sc-7984-R; Santa Cruz, CA [40])/rabbit monoclonal antibody against p-4E-BP1 (Thr37/46, 1:1000; #2855; Cell Signaling Technology, MA)/rabbit polyclonal antibody against Actin (I-19, 1:1000; sc-1616; Santa Cruz, CA). The membranes were then incubated with horseradish peroxidase-conjugated secondary antibodies (1:10000; KC-RB-035; KangCheng Biotechnology, China) for 2 h at room temperature. Immunoblotting signals were visualized by treatment with ECL reagent (WBKLS0050; Millipore Bioscience Research Reagents) and exposed to film. The bands were captured with the image analysis system and quantified using Quantity One software (Bio-Red laboratory). All bands were normalized relative to the corresponding β-actin band.

Inevitably, the antibody against p-4E-BP1 (#2855; Cell Signaling Technology, MA) may cross-react with 4E-BP2 and 4E-BP3 when phosphorylated at equivalent sites.

All histochemical and blotting data were calculated by an observer blind for treatment.

### Statistical analysis

All results were expressed as mean ± S.E.M. (standard error of the mean). Data of spontaneous response behaviors and western blots between each groups were compared by Owo-way ANOVA followed by a Bonferroni’s post hoc test. Two-way ANOVA followed by a Bonferroni’s post hoc test was used to analyze the effect of rapamycin or CCI-779 on the PWTL and PWMT values after BmK I administration.

## Results

### Unilateral injection of BmK I activates mTOR, p70 S6K and 4E-BP1 in bilateral spinal cord dorsal horn

**Figure 1:**
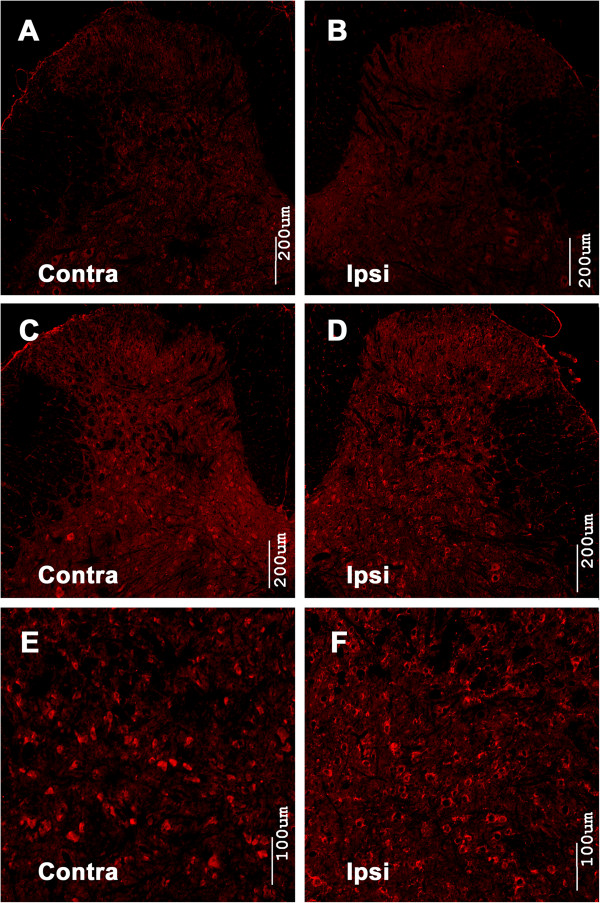
**Immunoreactivity of p-mTOR in the spinal cord dorsal horn after intraplantar BmK I injection.** Compared with the saline group **(A**, **B)**, BmK I treated groups **(C**-**F)** showed obvious immunoreactivity of p-mTOR in both ipsi- and contralateral spinal cord dorsal horn.

**Figure 2:**
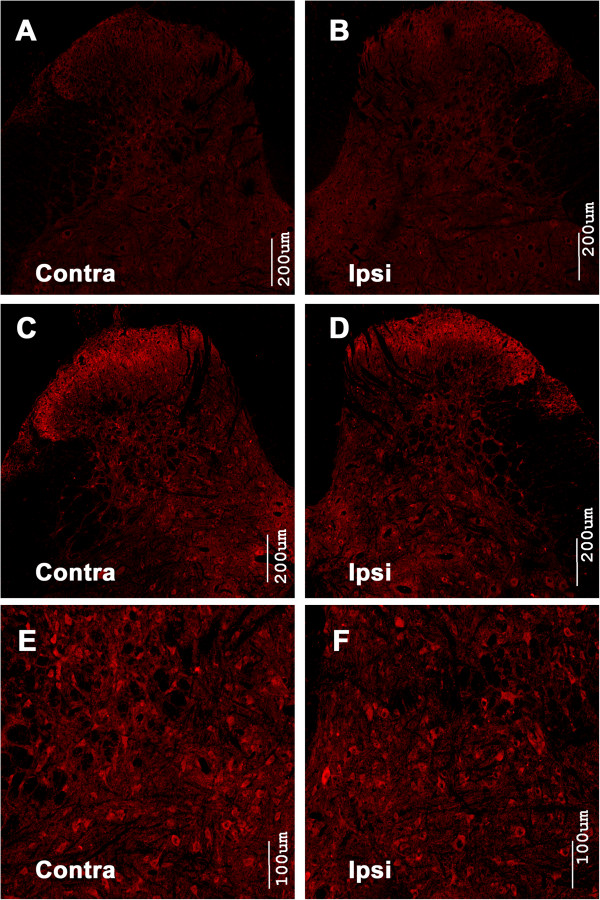
**4E-BP1 is phosphorylated in the spinal cord dorsal horn after intraplantar BmK I injection.** Compared with the saline group **(A**, **B)**, BmK I treated groups **(C**-**F)** showed obvious immunoreactivity of p-4E-BP1 in both ipsi- and contralateral spinal cord dorsal horn.

**Figure 3:**
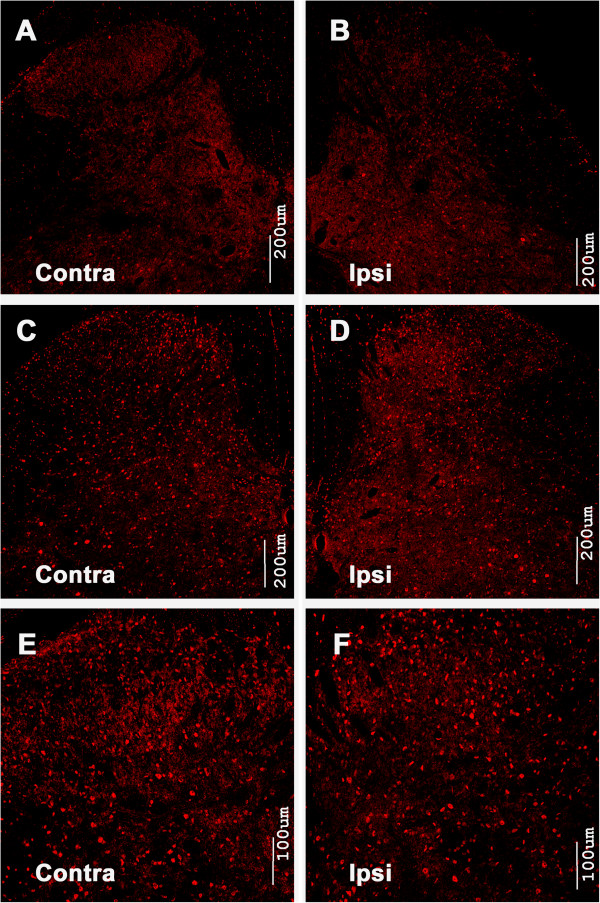
**Immunoreactivity of p-p70 S6K in the spinal cord dorsal horn after intraplantar BmK I injection.** Compared with the saline group **(A**, **B)**, the immunoreactivity of p-p70 S6K in both sides of spinal cord dorsal horn increased significantly in BmK I treated groups **(C**-**F)**.

Immunostaining was performed to evaluate whether the level of phosphorylated mTOR, p70 S6K and 4E-BP1 had changed following BmK I administration. In saline injected rats, the intensity of p-mTOR immunostaining in the spinal cord was low (Figure 1A-B). Two hours following BmK I injection, p-mTOR-immunoreactive cells were detected in abundance on both sides of the spinal cord dorsal horn. The p-mTOR labeled cells was observed in all dorsal horn laminas, particularly in the deep layers (lamina IV–VI) (Figure 1C-F). Levels of mTOR, p-4E-BP1 and p-p70 S6K showed similar trends. After BmK I administration, p-4E-BP1 reactivity increased in the superficial layers (Lamina I-II) and deep layers (Lamina IV-VI) (Figure 2), meanwhile p-p70 S6K positive signals increased markedly in all laminas of the spinal cord dorsal horn (Figure 3).

**Figure 4:**
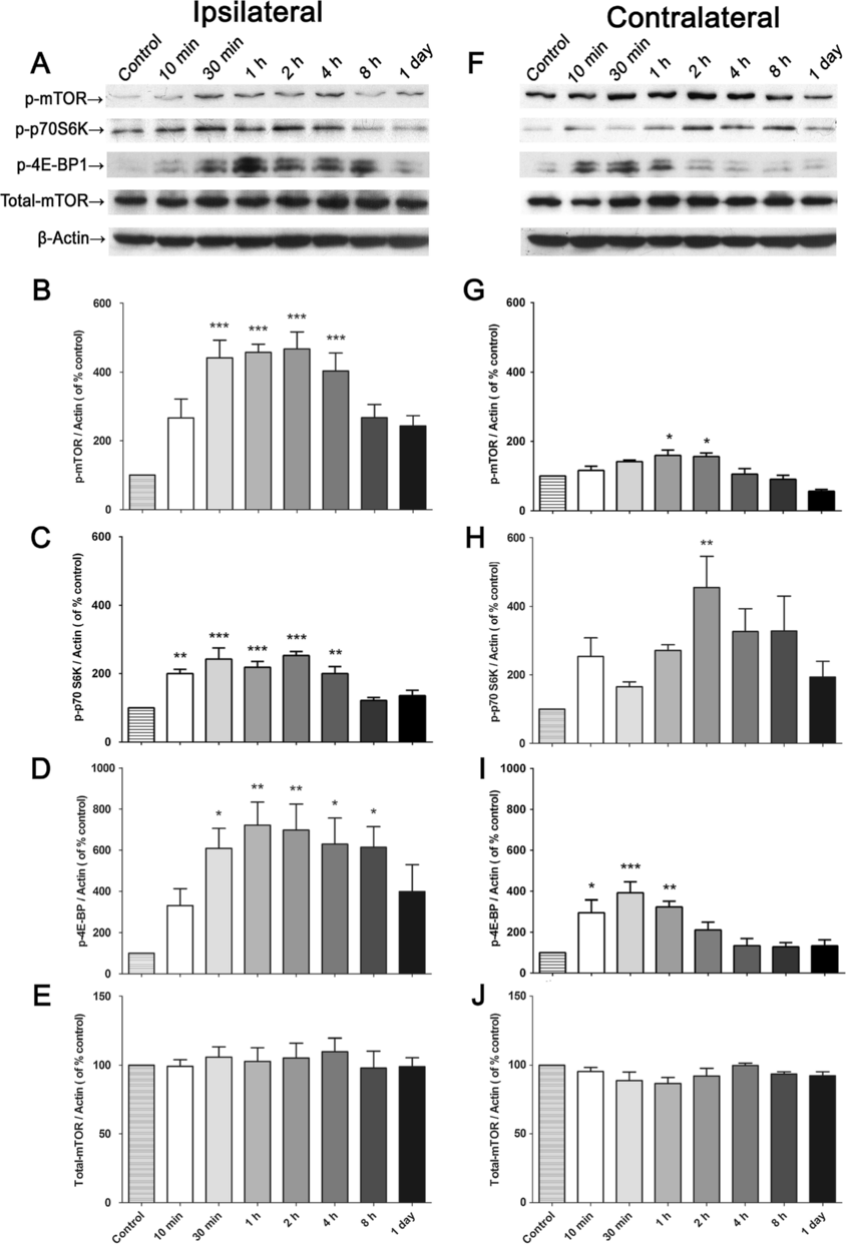
**BmK I-induced changes in spinal levels of p-mTOR, p-4E-BP1 and p-p70 S6K assessed by Western blot. (A)** &**(F)**, representative western blots showing levels of p-mTOR, p-4E-BP, p-p70 S6K, total mTOR and β-actin in both ipsi- **(A)** and contralateral **(B)** side of the spinal cord. **(B**-**E)** &**(G**-**J)**, histograms represent the mean levels with respect to each control group at different time points after intraplantar BmK I injection. The data are presented as mean ± S.E.M. of three rats per group. *p<0.05, **p<0.01, ***p<0.001, compared with control group by One-way ANOVA, followed by Bonferroni's post hoc test.

In addition, western blotting experiments were performed to confirm the immunostaining results. In naive rats, limited activation of mTOR, as well as 4E-BP1 and p70 S6K, was found on each side of the spinal cord. In response to BmK I injection, the amount of p-mTOR on the injured side increased rapidly and then peaked at 2 h (Figure 4B). The quantity of p-p70 S6K and p-4E-BP1 was noted to rise as well and returned to baseline at 1 day (Figure 4C-D). Compared to the ipsilateral side, a slighter but notable activation of the above molecules was observed in the contralateral side (Figure 4F-I). The total amount of mTOR was not affected by BmK I treatment (Figure 4E and J).

### Cellular localization of phosphorylated mTOR, p70 S6K and 4E-BP1 in spinal cord dorsal horn

In order to determine which types of spinal cells were involved in mTOR-mediated pain-related behaviors induced by BmK I, double-label immunohistochemical staining was performed.

**Figure 5:**
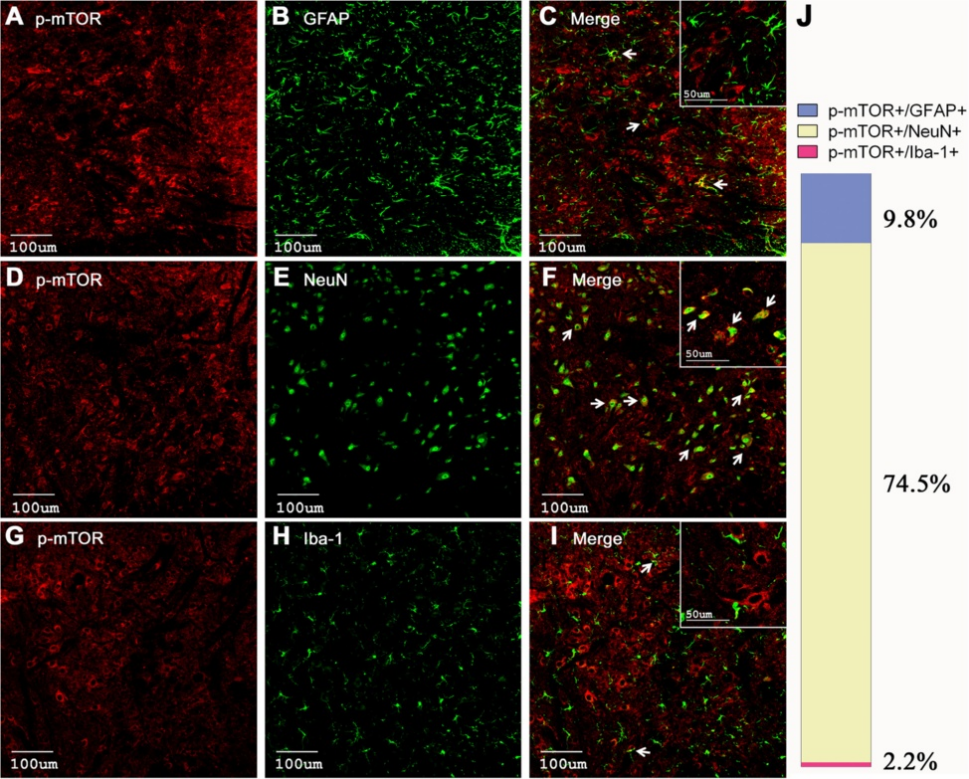
**Cellular localization of p-mTOR immunoreactivity in the spinal cord dorsal horn. (A-I)** Cell-type-specific immunolabeling of p-mTOR in the ipsilateral dorsal horn at 2 h after intraplantar BmK I injection. Arrows indicate colocalization of the p-mTOR (red) with the respective cell markers (green). **(J)** Histogram of the cellular distribution of p-mTOR.

Confocal microphotography revealed that 74.5 ± 4.2% p-mTOR immunostaining positive profiles were co-localized with the neuronal marker NeuN in the dorsal horn (Figure 5D-F). p-mTOR could be detected in only 9.8 ± 1.2% GFAP-positive astrocytes (Figure 5A-C) and 2.2 ± 0.7% Iba-1-positive microglia (Figure 5G-I).

**Figure 6:**
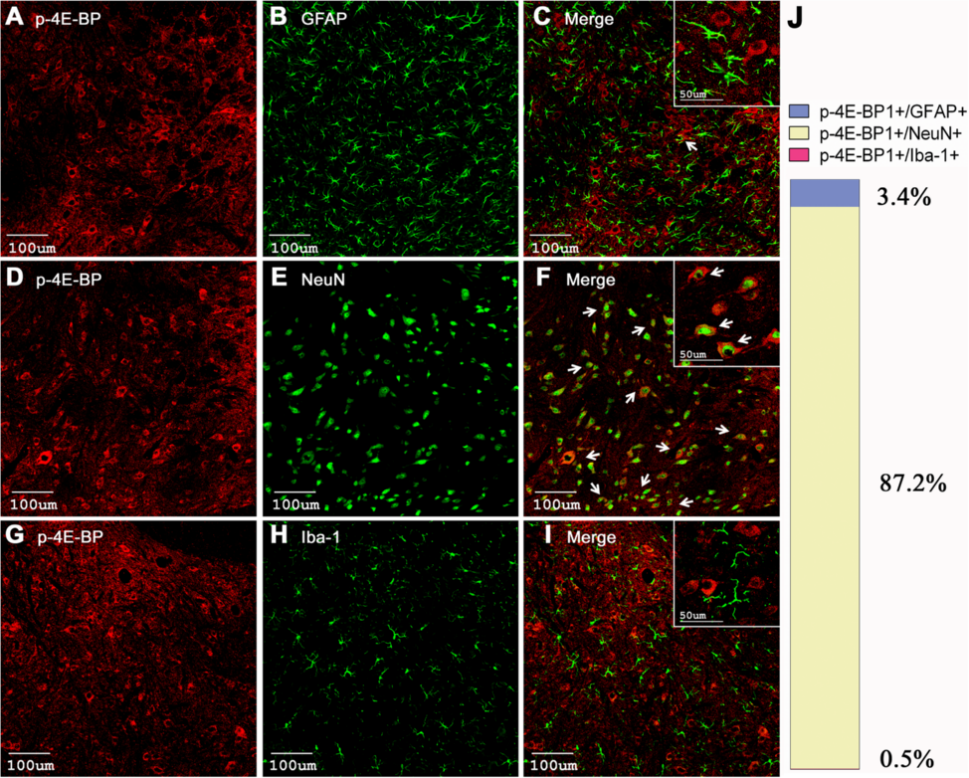
**Cellular localization of p-4E-BP1 immunoreactivity in the spinal cord dorsal horn. (A**-**I)** Cell-type-specific immunolabeling of p-4E-BP1 in the ipsilateral dorsal horn at 2 h after intraplantar BmK I. Arrows indicate colocalization (yellow) of the p-4E-BP1 (red) with the respective cell markers (green). **(J)** Statistical histogram of the cellular distribution of p-4E-BP1.

**Figure 7:**
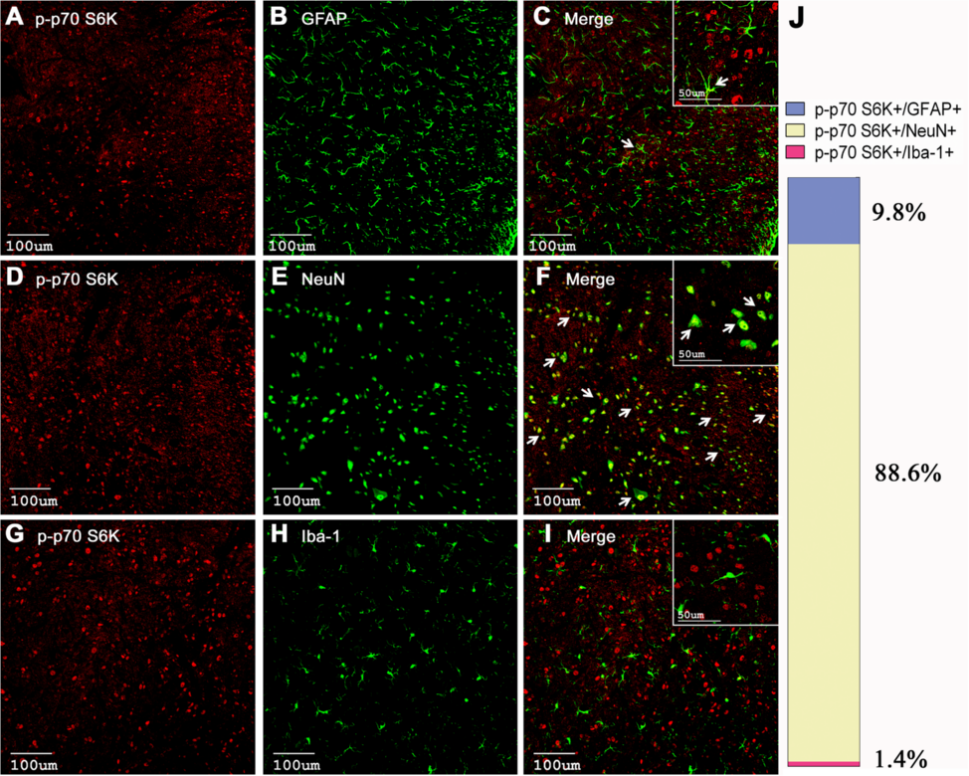
**Cellular localization of p-p70 S6K immunoreactivity in the ipsilateral spinal cord dorsal horn. (A**-**I)** Cell-type-specific immunolabeling of p-p70 S6K in the ipsilateral dorsal horn at 2 h after intraplantar BmK I. Arrows indicate colocalization (yellow) of the p-p70 S6K (red) with the respective cell markers (green). **(J)** Statistical histogram of the cellular distribution of p-p70 S6K.

Moreover, p-4E-BP1 was mainly observed in the spinal neurons (87.2 ± 1.6%, Figure 6D-F) and only a few light signals could be detected in astrocytes (3.4 ± 0.4%, Figure 6A-C), but not in microglia (Figure 6G-I). The p-p70 S6K-positive cells were co-localized with spinal neurons (88.6 ± 5.3%, Figure 7D-F) but scarcely with microglia (Figure 7A-B).

### Inhibiting spinal mTOR attenuates BmK I-induced rat pain responses

Rapamycin and CCI-779, two specific inhibitors of mTOR [23], were employed to investigate the potential involvement of the mTOR pathway in BmK I-induced pain-related behaviors.

**Figure 8:**
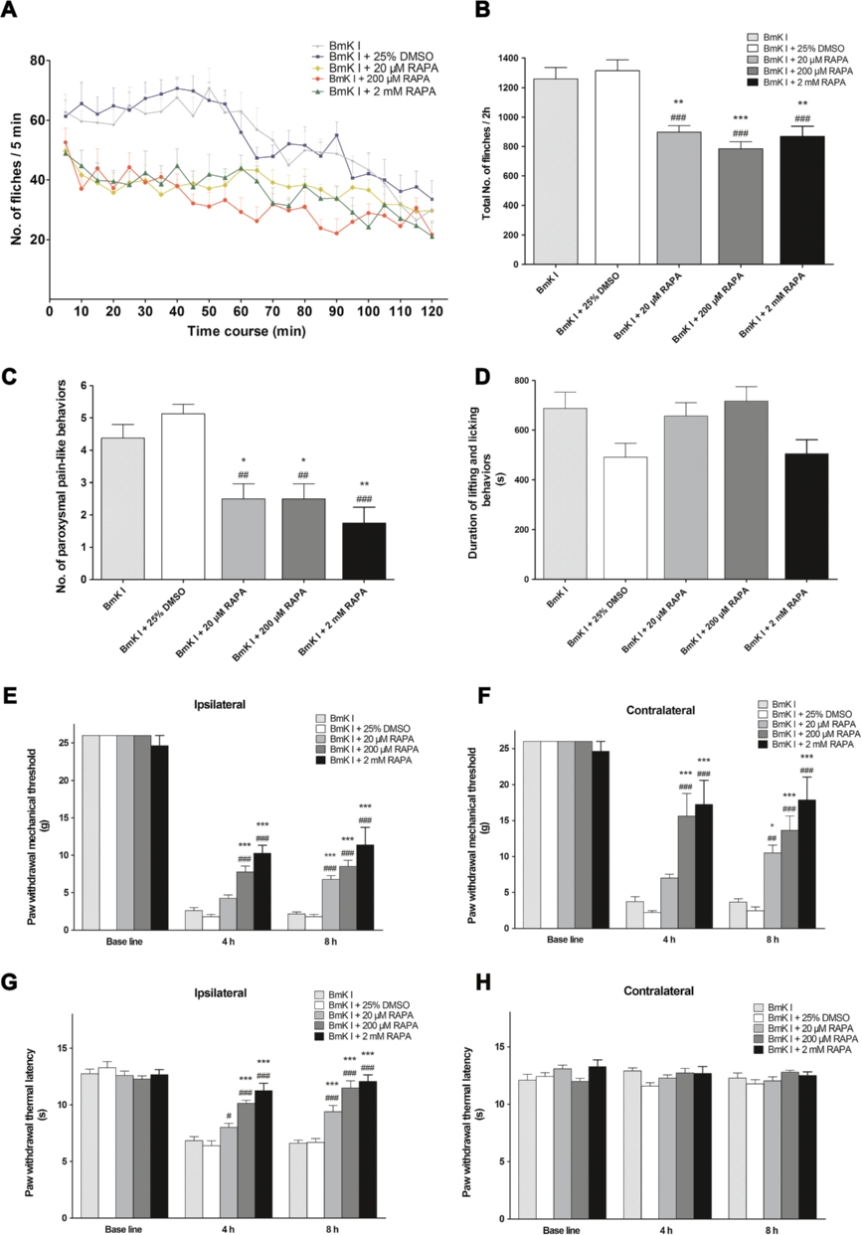
**Suppression effect of rapamycin on BmK I-induced rat pain behaviors. (A)** Rat flinch behavior was attenuated when rats were pre-treated with rapamycin (20 μM, 200 μM and 2 mM) 30 min before BmK I administration. Suppression of total number of the rat paw flinches **(B)** and total number of paroxysmal pain-like behaviors **(C)** by rapamycin (20 μM, 200 μM and 2 mM) during 2 h after BmK I injection. Suppression effect on ipsilateral **(E)** and contralateral **(F)** mechanical hypersensitivity, and ipsilateral **(G)** thermal hypersensitivity by different doses of rapamycin pre-treatment (20 μM, 200 μM and 2 mM). However, rapamycin did NOT affect the lifting and licking behaviors **(D)** and the contralateral basal thermal threshold value **(H)**. *p<0.05, **p<0.01, ***p<0.001, compared with BmK I group; #*p*<0.05, ##*p*<0.01, ###*p*<0.001, compared with DMSO vehicle group. n=8 for each group.

Rapamycin or vehicle was i.t. injected at 30 min prior to BmK I administration. Diluted DMSO, as vehicle, did not affect BmK I-induced pain-related spontaneous behaviors in rats. Compared with BmK I group or the vehicle group, rapamycin (20 μM, 200 μM or 2 mM) suppressed the spontaneous flinching behaviors significantly (Figure 8A, Table 1). The suppression of rapamycin on flinches lasted for at least 90 min. Pre-treatment with rapamycin at doses of 20 μM, 200 μM and 2 mM decreased the total number of flinches and the number of paroxysmal pain-like behaviors induced by BmK I within 2 h (Figure 8B-C). However, the time duration of lifting and licking behaviors was not affected by rapamycin (Figure 8D). Furthermore, BmK I-induced hypersensitivity is influenced by rapamycin. Bilateral mechanical hypersensitivity and ipsilateral thermal hypersensitivity were dose-dependently suppressed by pre-treatment with rapamycin at the time point of 4 h and 8 h (Figure 8E-G, Table 2).

**Table 1:** The No. of rat flinch behavior in respective groups

	**BmK I**	**BmK I+25%DMSO**	**BmK I+20 μM RAPA**	**BmK I+200 μM RAPA**	**BmK I+2 mM RAPA**
**5 min**	62.8±6.0	61.4±5.3	49.8±2.8	52.6±4.8	48.9±3.7
**10 min**	59.7±3.6	65.6±7.2	41.8±3.0 #	37.0±4.0 *, ##	44.8±5.2 #
**15 min**	59.3±3.6	62.1±5.7	39.0±5.0 #	43.9±6.6	39.9±5.8
**20 min**	58.6±5.0	64.9±6.1	35.8±4.0 **, ##	37.3±5.2 *, ##	39.5±3.7 *, ##
**25 min**	65.1±6.1	63.5±6.0	39.1±5.2 **, #	44.3±5.0	38.4±4.5 **, #
**30 min**	62.3±6.9	67.4±5.5	39.9±5.1	39.1±2.8 #	42.4±7.4 #
**35 min**	63.0±4.4	68.9±4.8	35.1±3.5 ***, ###	41.0±1.5 *, ###	38.6±6.2 **, ###
**40 min**	67.7±7.0	70.8±3.3	37.9±2.2 **, ###	38.0±4.0 **, ###	44.8±5.8 *, ##
**45 min**	61.6±6.2	69.9±5.0	38.9±2.4 **, ###	32.1±3.1 ***, ###	37.6±5.4 **, ###
**50 min**	70.9±6.7	66.8±6.1	37.1±3.1 **, ##	31.1±4.8 ***, ###	41.9±6.7 *, #
**55 min**	62.7±5.7	65.5±4.7	38.3±4.4 **, ##	33.3±6.1 ***, ###	40.8±3.0 *, #
**60 min**	63.1±6.3	56.0±6.3	43.5±1.8	29.4±4.6 ***, ##	44.0±4.3
**65 min**	57.0±7.2	47.4±3.6	43.3±2.8	26.3±4.8 **, #	39.6±5.1
**70 min**	53.4±2.8	47.8±3.9	39.1±4.3	31.8±3.7 *	32.3±5.2 *
**75 min**	45.0±2.0	52.1±4.5	37.6±4.0	29.9±3.5 *, ###	31.4±2.8 *, ##
**80 min**	50.0±6.2	51.6±6.3	38.4±5.4	31.0±5.2 **, ###	38.0±3.2
**85 min**	49.3±4.5	48.0±4.7	36.8±1.8 #	23.9±3.0 **, ###	33.8±3.8 #
**90 min**	48.9±5.2	55.0±4.6	33.5±4.0	22.1±5.0	33.9±4.2
**95 min**	46.4±1.8	40.6±5.9	37.5±5.0	26.0±5.3	29.3±4.8 *
**100 min**	43.4±5.3	42.1±7.0	36.6±3.5	29.0±3.0	24.3±4.2
**105 min**	39.0±2.7	40.0±6.9	31.8±3.5	28.1±4.0	31.6±6.3
**110 min**	31.7±4.4	36.1±5.5	31.8±2.6	24.6±2.7	27.1±5.4
**115 min**	26.6±2.8	37.6±5.3	29.5±3.3	30.6±3.4	24.8±5.2
**120 min**	30.1±5.6	33.5±6.3	29.8±3.8	21.8±3.9	21.1±5.2
**Total**	1258.8±76.7	1314.63±72.71	896.63 ± 44.46 **, ###	784.00 ± 48.79 ***, ###	868.38 ± 68.76 **,###

Note: *p<0.05, **p<0.01, ***p<0.001, compared with BmK I group. #p<0.05, ##p<0.01, ###p<0.001, compared with DMSO group.

One-way ANOVA followed by a Bonferroni's post hoc test.

**Table 2:** Effect of rapamycin (pre-treatment) on mechanical and thermal hypersensitivity induced by BmK I

	**Paw withdrawal mechanical threshold (g)**					**Paw withdrawal thermal latency (s)**				
	**Naive**	**Vehicle**	**Rapa**	**Rapa**	**Rapa**	**Naive**	**Vehicle**	**Rapa**	**Rapa**	**Rapa**
			**20 μM**	**200 μM**	**2 mM**			**20 μM**	**200 μM**	**2 mM**
**Ipsilateral**										
**Base line**	26.00 ± 0.00	26.00 ± 0.00	26.00 ± 0.00	26.00 ± 0.00	24.63 ± 1.38	12.74 ± 0.32	13.28 ± 0.38	12.60 ± 0.27	12.26 ± 0.24	12.66 ± 0.37
**4 h**	2.625 ± 0.42	1.80 ± 0.32	4.25 ± 0.45	7.75 ± 0.80 ***, ###	10.25 ± 1.08 ***, ###	6.84 ± 0.26	6.38 ± 0.32	8.01 ± 0.27 #	10.13 ± 0.23 ***, ###	11.24 ± 0.51 ***, ###
**8 h**	2.18 ± 0.27	1.8 ± 0.32	6.75 ± 0.53 ***, ###	8.5 ± 0.82 ***, ###	11.38 ± 2.35 ***, ###	6.62 ± 0.23	6.69 ± 0.29	9.39 ± 0.39 ***, ###	11.48 ± 0.55 ***, ###	12.06 ± 0.48 ***, ###
**Contralateral**										
**Base line**	26.00 ± 0.00	26.00 ± 0.00	26.00 ± 0.00	26.00 ± 0.00	24.63 ± 1.38	12.10 ± 0.43	12.43 ± 0.26	13.09 ± 0.26	11.99 ± 0.23	13.28 ± 0.49
**4 h**	3.75 ± 0.70	2.25 ± 0.25	7 ± 0.53	15.63 ± 3.17 ***, ###	17.25 ± 3.38 ***, ###	12.9 ± 0.20	11.58 ± 0.23	12.26 ± 0.24	12.71 ± 0.37	12.69 ± 0.49
**8 h**	3.68 ± 0.50	2.50 ± 0.50	10.50 ± 1.10 *, ##	13.63 ± 2.03 ***, ###	17.88 ± 3.20 ***, ###	12.28 ± 0.33	11.78 ± 0.28	12.04 ± 0.29	12.79 ± 0.12	12.50 ± 0.23

Note: *p<0.05, **p<0.01, ***p<0.001, compared with BmK I group; #p<0.05, ##p<0.01, ###p<0.001, compared with vehicle group. Two-way ANOVA followed by a Bonferroni's post hoc test.

**Figure 9:**
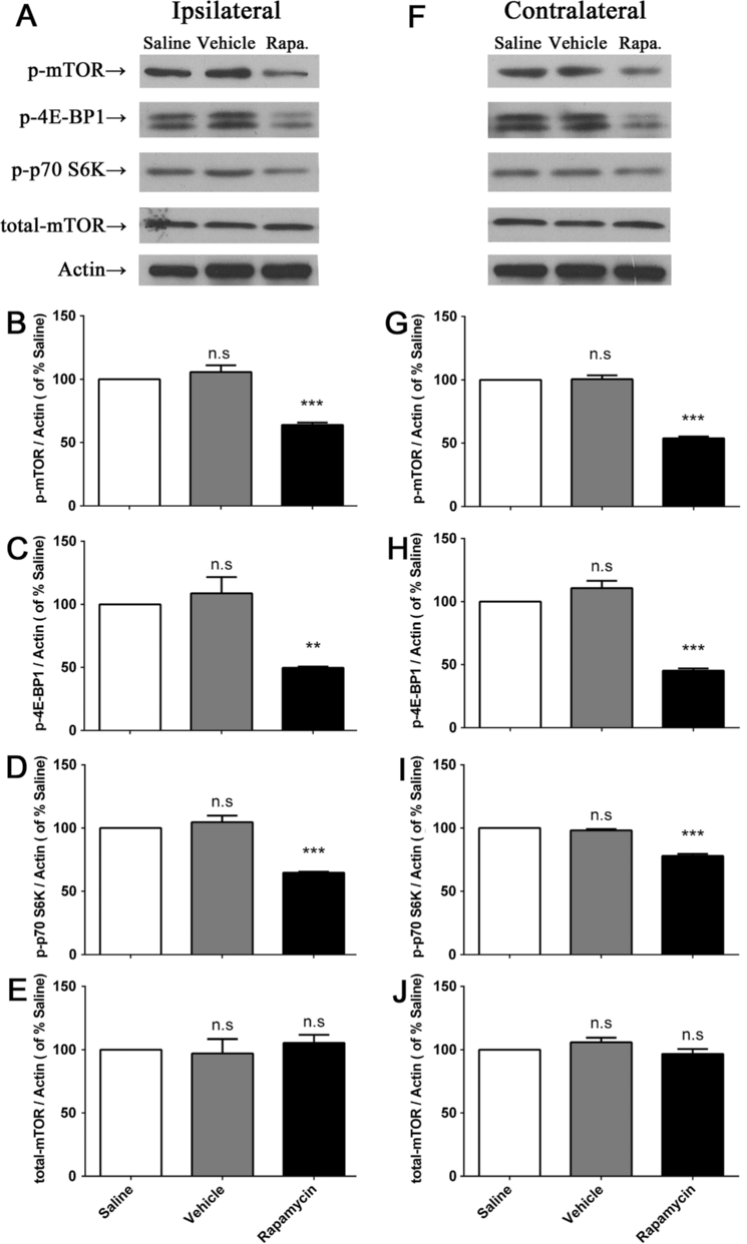
**200 μM rapamycin inhibited the activation of spinal mTOR, 4E-BP1 and p70 S6K assessed by Western blot. (A)** &**(F)**, representative western blots showing levels of p-mTOR, p-4E-BP, p-p70 S6K, total mTOR and β-actin in ipsi- **(A)** and contralateral **(B)** side of the spinal cord. **(B**-**E)** &**(G**-**J)**, histograms represent the mean levels with respect to each saline-treated group at 2 h after i.pl. BmK I injection. The data are presented as mean ± S.E.M. of three rats per group. N.s., **p<0.01, ***p<0.001, compared with saline-treated group by One-way ANOVA, followed by Bonferroni's post hoc test.

Western blots revealed a significant inhibition of 200 μM rapamycin on mTOR cascades in dorsal spinal cord compared with vehicle and saline (Figure 9). I.t. rapamycin not only decreased the activation of p-mTOR (Figure 9B & G) in both sides, but also p-4E-BP1 (Figure 9C & H) and p-p70 S6K (Figure 9D & I). The total amount of mTOR was not affected by rapamycin.

**Figure 10:**
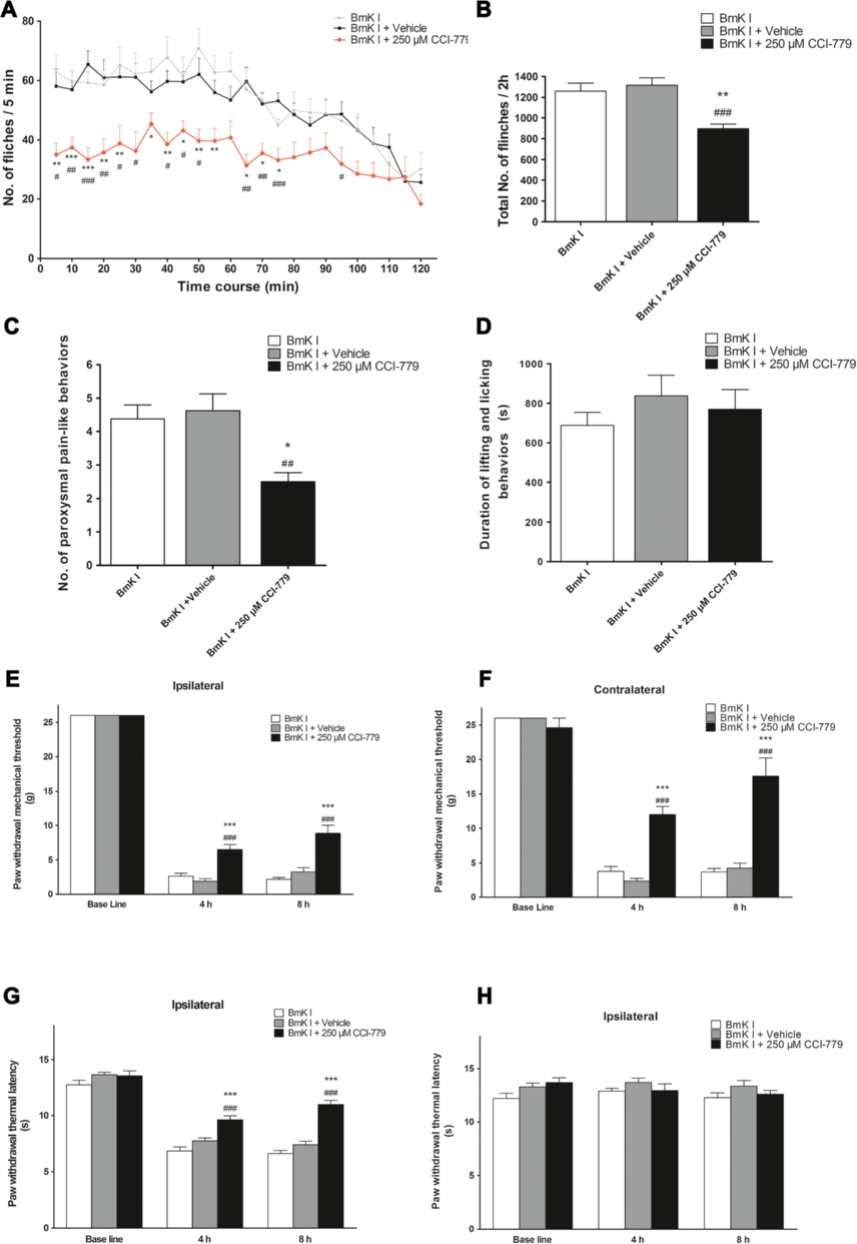
**Suppression effect of CCI-779 on BmK I-induced rat pain behaviors. (A)** Rat flinch behavior was attenuated when rats were pre-treated with CCI-779 (250 μM) 30 min before BmK I administration. Suppression of total number of the rat paw flinches **(B)** and total number of paroxysmal pain-like behaviors **(C)** by CCI-779 during 2 h after BmK I injection. **(D)** No effects on lifting and licking behaviors. Suppression on ipsilateral **(E)** and contralateral **(F)** mechanical, and ipsilateral thermal **(G)** hypersensitivity by CCI-779, but no effect on contralateral basal thermal threshold value **(H)**. *p<0.05, **p<0.01, ***p<0.001, compared with BmK I group. #*p*<0.05, ##*p*<0.01, ###*p*<0.001, compared with vehicle group. n=8 for each group.

Likewise, CCI-779 showed remarkable inhibitory effect on BmK I-induced spontaneous responses (Figure 10A-C) and hypersensitivity (Figure 10E-G, Table 3). A 250 μM CCI-779 pre-treatment decreased the total number of flinches (Figure. 10B) and the number of paroxysmal pain-like behaviors (Figure 10C) within 2 h, inhibited BmK I-induced bilateral mechanical hypersensitivity and ipsilateral thermal hypersensitivity efficiently (Figure 10E-H, Table 3). As well, CCI-779 was incapable of suppressing lifting and licking behaviors (Figure 10D).

**Table 3:** Effect of CCI-779 (pre-treatment) on mechanical and thermal hypersensitivity induced by BmK I

	**Paw withdrawal mechanical threshold (g)**			**Paw withdrawal thermal latency (s)**		
	**Naïve**	**Vehicle**	**CCI-779**	**Naïve**	**Vehicle**	**CCI-779**
**Ipsilateral**						
**Base line**	26.00 ± 0.00	26.00 ± 0.00	26.00 ± 0.00	12.74 ± 0.40	13.64 ± 0.23	13.55 ± 0.44
**4 h**	2.63 ± 0.42	1.88 ± 0.36	6.50 ± 0.73 ***, ###	6.84 ± 0.35	7.75 ± 0.26	9.64 ± 0.35 ***, ###
**8 h**	2.18 ± 0.27	3.25 ± 0.62	8.88 ± 1.16 ***, ###	6.62 ± 0.28	7.43 ± 0.30	10.99 ± 0.37 ***, ###
**Contralateral**						
**Base line**	26.00 ± 0.00	26.00 ± 0.00	24.63 ± 1.38	12.10 ± 0.50	13.28 ± 0.37	13.69 ± 0.44
**4 h**	3.75 ± 0.70	2.35 ± 0.37	12.00 ± 1.17 ***, ###	12.90 ± 0.27	13.71 ± 0.39	12.95 ± 0.61
**8 h**	3.68 ± 0.50	4.25 ± 0.70	17.63 ± 2.61 ***, ###	12.28 ± 0.45	13.36 ± 0.53	12.61 ± 0.35

Note: ***p<0.001, compared with BmK I group; ###p<0.001, compared with cehicle group. Two-way ANOVA, Bonferroni's post hoc test.

### Post-treatment with rapamycin attenuate BmK I-induced hyperalgesia

**Figure 11:**
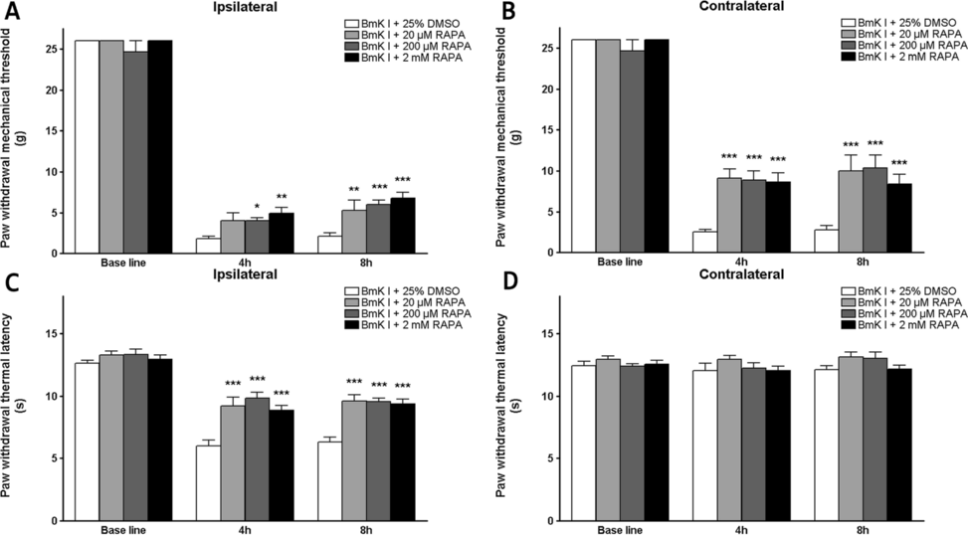
**Post-treatment with rapamycin suppressed unilateral thermal hypersensitivity and bilateral mechanical hypersensitivity induced by intraplantar BmK I injection. (A** and **B)** showed the suppressive effects on BmK I-induced the ipsilateral **(A)** and contralateral **(B)** mechanical hypersensitivity by different doses of rapamycin post-treatment (20 μM, 200 μM and 2 mM). **(C** and **D)** showed that different doses of rapamycin post-treatment (20 μM, 200 μM and 2 mM) suppressed ipsilateral **(C)** thermal hypersensitivity but did not affect the contralateral basal thermal threshold value **(D)**. **P<0.01, ***P<0.001, compared with relevant control groups treated with DMSO. n=8 for each group.

Post-treatment with rapamycin was also found to be able to attenuate BmK I-induced hypersensitivity. Bilateral mechanical hypersensitivity and unilateral thermal hypersensitivity could be suppressed by post-treatment with rapamycin (20 μM, 200 μM and 2 mM) significantly (Figure 11A-C, Table 4). Neither pre- nor post-treatment of rapamycin altered the contralateral basal PWTL values (Figure 9H; Figure 11D).

**Table 4:** Effect of rapamycin (post-treatment) on mechanical and thermal hypersensitivity induced by BmK I

	**Paw withdrawal mechanical threshold (g)**				**Paw withdrawal thermal latency (s)**			
	**Vehicle**	**Rapa**	**Rapa**	**Rapa**	**Vehicle**	**Rapa**	**Rapa**	**Rapa**
		**20 μM**	**200 μM**	**2 mM**		**20 μM**	**200 μM**	**2 mM**
**Ipsilateral**								
**Base line**	26.00 ± 0.00	26.00 ± 0.00	24.63 ± 1.38	26.00 ± 0.00	12.59 ± 0.19	13.28 ± 0.25	13.30 ± 0.37	12.94 ± 0.27
**4 h**	1.80 ± 0.21	4.00 ± 0.89	4.00 ± 0.18 *	4.93 ± 0.56 **	5.98 ± 0.36	9.21 ± 0.60 ***	9.83 ± 0.39 ***	8.86 ± 0.28 ***
**8 h**	2.13 ± 0.33	5.25 ± 1.08 **	6.00 ± 0.35 ***	6.75 ± 0.62 ***	6.36 ± 0.31	9.61 ± 0.41 ***	9.55 ± 0.22 ***	9.38 ± 0.12 ***
**Contralateral**								
**Base line**	26.00 ± 0.00	26.00 ± 0.00	24.63 ± 1.38	26.00 ± 0.00	12.43 ± 0.26	12.94 ± 0.23	12.36 ± 0.16	12.54 ± 0.24
**4 h**	2.50 ± 0.27	9.13 ± 0.83 ***	8.88 ± 0.74 ***	8.63 ± 0.81 ***	12.04 ± 0.42	12.94 ± 0.21	12.21 ± 0.34	12.03 ± 0.23
**8 h**	2.75 ± 0.40	10.00 ± 1.77 ***	10.38 ± 1.23 ***	8.38 ± 0.87 ***	12.11 ± 0.25	13.1 ± 0.35	13 ± 0.36	12.13 ± 0.21

Note: *p<0.05, **p<0.01, ***p<0.001, compared with relevant vehicle groups. Two-way ANOVA followed by a Bonferroni's post hoc test.

## Discussion

### The localization of mTOR cascades in the spinal cord under pain condition

In the physiological condition, the level of phosphorylated mTOR, 4E-BP1/2 and p70 S6K were extremely low and they were only detected in spinal cord dorsal horn neurons, but not in astrocytes or microglia [29]. In the present study, we found that the level of phosphorylated mTOR, p70 S6K and 4E-BP1 increased significantly following BmK I injection, which were predominantly observed in spinal neurons. Nevertheless, a small amount of positive signals were detected in astrocyte (Figure 5–7). This result is consistent with previous reports from the formalin test and carrageenan-induced pain model, where it was observed that p-mTOR and p-4E-BP1 exist in spinal neurons and astrocytes [31].

Astrocytes are believed to contribute to the maintenance of chronic pain since the activation of astrocytes is delayed under conditions of peripheral injury [41,42]. In our previous studies, astrocytes were demonstrated to contribute to BmK sting-induced nociceptive behaviors. The activation of astrocytes in bilateral spinal cord initiated from day 3, peaked at day 7 following i.pl. injection of BmK venom [43]. Although there is still no direct evidence on the relationship between pain processing and mTOR activation in spinal glia, some studies have discovered valuable information. Codeluppi et al. found that mTOR might mediate the astrocytic inflammatory reaction after nerve injury [44]. Tezel et al. recently reported that, accompanying the development of inflammatory responses induced by experimental glaucoma, mTOR signaling pathway was activated [45]. Integrating these investigation, whether we can approach a supposition that the astroglial activation of mTOR pathway contribute to the neuronal sensitization via control the release of inflammatory factors? It needs further demonstration.

In BmK I-induced pain, we did not find high amounts of p-p70 S6K was co-labeled with glial cells. Moreover, capsaicin and carrageenan induced activation of S6 protein (the substrate of p70 S6K) was found primarily in spinal neurons but not glial cells, which is confirmed by the results presented [46,47]. However, in some studies carrageenan ignited spinal p-S6 was also detected in microglia [31]. In fact, carrageenan induced microglial activation of S6 is beyond overlapped with p-mTOR and the co-labeling of p-S6 and microglia was extremely few. This may suggest the existence of other signaling pathways other than mTOR/p70 S6K that can result in phosphorylated S6 and indicate that whether p70 S6K or S6 protein was activated in microglia is irrelevant to the development of pain sensation. Also, the early activation of downstream components before mTOR activation might be owing to the intracellular pathways, including ERK1/2, PKB and PKC. Shin et al. found that glycogen synthase kinase (GSK)-3 was another up-stream modulator of p70 S6K except mTOR [48]. Even so, mTOR could be still served as the predominant modulator while other factors might play minor roles. It could be verified by the pharmacological and behavioral tests: pre-administration of rapamycin, the specific inhibitor of mTOR, significantly suppressed the activation of mTOR, as well as p70 S6K and 4E-BP1 (Figure 9), which attenuating pain-related behaviors accordingly (Figure 8).

### The activation of mTOR cascades in different animal pain models

Although, most of previous studies supported the view that mTOR cascades were involved in pain sensation, the opinion was still conflicting regarding to the activation modes in different animal models. Géranton et al. reported that activated mTOR in SNI model was found in a subpopulation of A-fiber dorsal root ganglion neurons and in spinal lamina I/III projection neurons [46], while Asante et al. found activated mTOR pathway components in some C-fiber dorsal root ganglion neurons and in spinal lamina II interneurons [49]. Here we mimicked a scorpion stinging-induced chronic pain state through employment of BmK I. Activated mTOR and its downstream components could be found in all spinal laminae in the model. Moreover, another recent study suggested that p-mTOR was co-labeled with 61.3±1.8% CGRP-positive C-fiber neurons, 64.6%±1.2% IB4-positive C-fiber neurons and 42.4%±1.2% NF200-positive A-fiber neurons in dorsal root ganglion after BmK I administration (unpublished data). Hence, it allowed us to infer that the varied activation pattern of mTOR cascades might be owing to the different animal models used.

### The rapid behavioral anti-nociceptive effect of rapamycin / CCI-779

As with previous studies [30,31], a rapid anti-nociceptive effect of rapamycin on pain-related spontaneous behaviors was observed in the BmK I-induced pain model. The total number of flinches, paroxysmal pain-like behaviors and long-term pain hypersensitivity were significantly decreased following rapamycin/CCI-779 administration.

In the current study rapamycin (CCI-779 as well) has no effect on lifting and licking behavior induced by BmK I, whereas rapamycin significantly attenuated licking and biting but not on lifting and flinching elicited by formalin [30]. The seemingly diverse effects of rapamycin/CCI-779 might depend on the distinct characteristic of animal pain models. In the formalin test, the duration of licking behavior is greater than that of lifting. However, rats injected with BmK I spend more time on lifting but little time on biting and licking the injured hind paw. In summary, due to the absence of an effect on lifting behavior, mTOR inhibitors are not suppressors of the total lifting and licking behavior induced by BmK I.

### The possible relationship between mTOR-dependent signaling pathway and pain

Spinal synaptic plasticity is indispensable in the sensitization to peripheral noxious stimuli and generation of chronic pain [27]. Protein synthesis at the site of synapse and soma is required for the formation of neuronal plasticity [50,51]. Since the effects of mTOR on neuronal synaptic plasticity are at least partly related to the phosphorylation of 4E-BP1 and p70 S6K [52-54], two vital regulators of protein expression, it is reasonable to consider mTOR as an incontrovertible candidate participating in the modulation of nociceptive transmission in the central nervous system. Moreover, mTOR activity has been demonstrated to be necessary for the electric stimulation-evoked late-phase long-term potentiation (L-LTP) and BDNF-induced strengthening of synaptic transmission in acute rodent hippocampal slices [55]. Agonists of metabotropic glutamate receptors induced-LTD in hippocampal CA1 area could be blocked by rapamycin [56]. All the evidence suggests mTOR signaling pathway is of vital importance to the central plasticity. Behavioral testing has confirmed that mTOR-dependent spinal mRNA translation is responsible for formalin-induced spinal neuronal hyperexcitability and carrageenan-induced behavioral hyperalagesia [30,47,57]. Apart from controlling protein synthesis, we have to note that mTOR is implicated in multiple processes, including ubiquitin-dependent proteolysis, microtubule and actin dynamics, all of which are crucial for regulating synaptic strength [15].

### Pre- and post-treatment with rapamycin decreased BmK I-induced behavioral hypersensitivity

The administration method of a therapeutic agent/molecule is a key factor of consideration. Clinically, the post-treatment phase is more relevant. Pre- and post-administration of rapamycin has shown excellent anti-nociceptive effect on both electrophysiological and behavioral studies in spared nerve injury (SNI) and formalin model [31,46,49]. In our research, the anti-nociceptive effect of pre- and post-treatment of rapamycin were evaluated in BmK I-induced pain model. After pre-treatment with rapamycin or CCI-779, the unilateral thermal hypersensitivity and bilateral mechanical hypersensitivity were significantly suppressed. This suggests that the mTOR signaling pathway mediates the induction of pain behaviors induced by BmK-I. Furthermore, rapamycin post-treatment also showed an effective anti-hypersensitivity effect. Thus, it is demonstrated that the mTOR-dependent signaling network was also involved in the maintenance of BmK I-induced pain.

However, the contribution of mTOR signaling pathway on thermal hyperalgesia is still controversial. While the mTOR inhibitor CCI-779 has no effect on spinal nerve ligation evoked thermal hypersensitivity [49], rapamycin exerted a significant inhibition on the responses to thermal stimuli in formalin and carrageenan-induced inflammatory pain [30,47]. What’s more, under short term inflammation pain state, nociceptive signals mediated by C-fiber and the responses of WDR neurons to the peripheral input was significantly inhibited by rapamycin [30]. In the present study, both rapamycin and CCI-779 efficiently increased the paw withdrawal thermal latency on BmK I-injected rats. It is thus clear that the inhibitors of mTOR are inclined to weaken inflammation but not nerve damage-induced thermal hypersensitivity. A possible determinant for this phenomenon might be the distinctive genesis of different animal models. Inflammatory pain is primarily attributed to inflammatory factors released from the inflamed tissue. Whereas neuropathic pain generally originates from the pathology of the nervous system. Neurogenic changes or injury leads to a steadier central and peripheral sensitization than that in inflammatory pain. Thus, the inhibitors of mTOR pathway are probably insufficient to weaken long term pathologic pain-induced thermal hypersensitivity.

In combination with previous results, including electrophysiological recording [7,58], behavioral observation [10], amino acid neurotransmitter release [12] and c-Fos expression in spinal cord dorsal horn [13], the potential mechanisms of BmK I-induced pain would rely firstly on the selective modulation of BmK I on voltage-gated sodium channels in the peripheral A and C primary afferent fibers, transmitting pain signals into the spinal cord, followed by an increase in spinal glutamate release and the activation of the neuronal mTOR signaling network. All these events lead to the elevated protein/peptides expression, and finally altered spinal plasticity.

## Conclusion

In summary, this work revealed that mTOR signaling pathway was dramatically activated on both sides of the rat spinal cord in response to the peripheral injection of BmK I. Both pre- and post-treatment of rapamycin showed a remarkable inhibition on spinal mTOR activity, and thereby displayed anti-nociceptive effect. The present study suggested that the mTOR-dependent signaling pathway, at least partially, contributes to BmK I-induced persistent pain. The mTOR signaling pathway might be a novel target to alleviate persistent and chronic pain.

## Competing interest

The authors declare that they have no conflict of interest.

## Authors’ contributions

FJ carried out the immunohistochemical staining, participated in western blotting, animal behavioral studies and drafted the manuscript. XYP participated in experimental design. QSN conceived and carried out behavioural experiments. LMH participated in western blotting. MC participated in immunohistochemical double labeling. YHJ conceived the project. All authors read and approved the final manuscript.

## Supplementary Material

Additional file 1: Figure S2 Immunostaining of p-mTOR was performed by using two individual antibodies from Cell Signaling Technology (CST, antibody 1 catalog #2976, antibody 2 catalog #2971). A&B, animal received i.pl. injection of saline and pre-administration of Rapamycin (i.t.). C&D, animal received i.pl. injection of saline and pre-administration of saline (i.t.). E&F, animal received i.pl. injection of BmK I and pre-administration of saline (i.t.). G&H, animal received i.pl. injection of BmK I and pre-administration of Rapamycin (i.t.). Click here for file

Additional file 2: Figure S3 Immunostaining of p-4E-BP1 was performed by using two individual antibodies: antibody 1 catalog BS5047 from Bioworld Technology and antibody 2 catalog #2855 from CST). A&B, animal received i.pl. injection of saline and pre-administration of Rapamycin (i.t.). C&D, animal received i.pl. injection of saline and pre-administration of saline (i.t.). E&F, animal received i.pl. injection of BmK I and pre-administration of saline (i.t.). G&H, animal received i.pl. injection of BmK I and pre-administration of Rapamycin (i.t.). Click here for file

Additional file 3: Figure S4 Activated p70 S6K was stained by two individual antibodies: antibody 1 catalog sc-7984-R from Santa Cruz and antibody 2 catalog #9204 from CST). A&B, animal received i.pl. injection of saline and pre-administration of Rapamycin (i.t.). C&D, animal received i.pl. injection of saline and pre-administration of saline (i.t.). E&F, animal received i.pl. injection of BmK I and pre-administration of saline (i.t.). G&H, animal received i.pl. injection of BmK I and pre-administration of Rapamycin (i.t.). Click here for file

Additional file 4: Figure S1 Illustration of quantitative assessment of double labeling staining. Selected (A) mTOR cascade positive microphotograph and (C) double labeled microphotograph (the same position as shown in A). (B) and (D) Software assisted evaluation of A & B: blue regions were defined as positive signals. (E) The measuring area, as well as the high magnified images in Figure 2, 3 and 4, was captured from lamina III-V of L5 spinal cord dorsal horn. Click here for file

## References

[B1] Balozet L Bucherl W, Buckley EE Scorpionism in the old world Venomous Animal and Their Venoms 1971 3 New York: Academic Press 349 371

[B2] Chen B Zhuo X Wang C Ji Y Asian scorpion BmK venom induces plasma extravasation and thermal hyperalgesia in the rat Toxicon 2002 40 527 533 10.1016/S0041-0101(01)00248-3 11821124

[B3] Bai ZT Liu T Chai ZF Pang XY Ji YH Rat pain-related responses induced by experimental scorpion BmK sting Eur J Pharmacol 2006 552 67 77 10.1016/j.ejphar.2006.09.018 17055482

[B4] Ji YH Mansuelle P Xu K Granier C Kopeyan C Terakawa S Rochat H Amino acid sequence of an excitatory insect-selective toxin (BmK IT) from venom of the scorpion Buthus martensi Karsch Sci China B 1994 37 42 49 8068186

[B5] Ji YH Wang WX Wang Q Huang YP The binding of BmK abT, a unique neurotoxin, to mammal brain and insect Na(+) channels using biosensor Eur J Pharmacol 2002 454 25 30 10.1016/S0014-2999(02)02363-4 12409001

[B6] Chai ZF Zhu MM Bai ZT Liu T Tan M Pang XY Ji YH Chinese-scorpion (Buthus martensi Karsch) toxin BmK alphaIV, a novel modulator of sodium channels: from genomic organization to functional analysis Biochem J 2006 399 445 453 10.1042/BJ20060035 16800812 PMC1615898

[B7] He H Liu Z Dong B Zhou J Zhu H Ji Y Molecular determination of selectivity of the site 3 modulator (BmK I) to sodium channels in the CNS: a clue to the importance of Nav1.6 in BmK I-induced neuronal hyperexcitability Biochem J 2010 431 289 298 10.1042/BJ20100517 20678086

[B8] Zhu MM Tao J Tan M Yang HT Ji YH U-shaped dose-dependent effects of BmK AS, a unique scorpion polypeptide toxin, on voltage-gated sodium channels Br J Pharmacol 2009 158 1895 1903 10.1111/j.1476-5381.2009.00471.x 19912232 PMC2807651

[B9] Ji YH Liu T The study of sodium channels involved in pain responses using specific modulators Sheng Li Xue Bao 2008 60 628 634 18958371

[B10] Bai ZT Liu T Jiang F Cheng M Pang XY Hua LM Shi J Zhou JJ Shu XQ Zhang JW Ji YH Phenotypes and peripheral mechanisms underlying inflammatory pain-related behaviors induced by BmK I, a modulator of sodium channels Exp Neurol 2010 226 159 172 10.1016/j.expneurol.2010.08.018 20736005

[B11] Liu ZR Ye P Ji YH Exploring the obscure profiles of pharmacological binding sites on voltage-gated sodium channels by BmK neurotoxins Protein Cell 2011 2 437 444 10.1007/s13238-011-1064-8 21748593 PMC4703586

[B12] Zhang XY Zhang JW Chen B Bai ZT Shen J Ji YH Dynamic determination and possible mechanism of amino acid transmitter release from rat spinal dorsal horn induced by the venom and a neurotoxin (BmK 1) of scorpion Buthus martensi Karsch Brain Res Bull 2002 58 27 31 10.1016/S0361-9230(02)00752-9 12121809

[B13] Bai ZT Zhang XY Ji YH Fos expression in rat spinal cord induced by peripheral injection of BmK I, an alpha-like scorpion neurotoxin Toxicol Appl Pharmacol 2003 192 78 85 10.1016/S0041-008X(03)00260-6 14554105

[B14] Sarbassov DD Ali SM Sabatini DM Growing roles for the mTOR pathway Curr Opin Cell Biol 2005 17 596 603 10.1016/j.ceb.2005.09.009 16226444

[B15] Jaworski J Sheng M The growing role of mTOR in neuronal development and plasticity Mol Neurobiol 2006 34 205 219 10.1385/MN:34:3:205 17308353

[B16] Kim DH Sarbassov DD Ali SM King JE Latek RR Erdjument-Bromage H Tempst P Sabatini DM mTOR interacts with raptor to form a nutrient-sensitive complex that signals to the cell growth machinery Cell 2002 110 163 175 10.1016/S0092-8674(02)00808-5 12150925

[B17] Kim D-H Sarbassov DD Ali SM Latek RR Guntur KVP Erdjument-Bromage H Tempst P Sabatini DM G^2^L, a Positive Regulator of the Rapamycin-Sensitive Pathway Required for the Nutrient-Sensitive Interaction between Raptor and mTOR Molecular cell 2003 11 895 904 10.1016/S1097-2765(03)00114-X 12718876

[B18] Peterson RT Schreiber SL Translation control: Connecting mitogens and the ribosome Curr Biol 1998 8 R248 10.1016/S0960-9822(98)70152-6 9545190

[B19] Gingras AC Gygi SP Raught B Polakiewicz RD Abraham RT Hoekstra MF Aebersold R Sonenberg N Regulation of 4E-BP1 phosphorylation: a novel two-step mechanism Genes Dev 1999 13 1422 1437 10.1101/gad.13.11.1422 10364159 PMC316780

[B20] Mothe-Satney I Brunn GJ McMahon LP Capaldo CT Abrahams RT Lawrence JC Mammalian target of rapamycin-dependent phosphorylation of PHAS-I in four (S/T)P sites detected by phospho-specific antibodies J Biol Chem 2000 275 33836 33843 10.1074/jbc.M006005200 10942774

[B21] Pause A Belsham GJ Gingras A-C Donze O Lin T-A Lawrence JC Sonenberg N Insulin-dependent stimulation of protein synthesis by phosphorylation of a regulator of 5’-cap function Nature 1994 371 762 767 10.1038/371762a0 7935836

[B22] Huang JX Manning BD A complex interplay between Akt, TSC2 and the two mTOR complexes Biochem Soc Trans 2009 37 217 222 10.1042/BST0370217 19143635 PMC2778026

[B23] Yang Q Guan KL Expanding mTOR signaling Cell Res 2007 17 666 681 10.1038/cr.2007.64 17680028

[B24] Jimenez-Diaz L Geranton SM Passmore GM Leith JL Fisher AS Berliocchi L Sivasubramaniam AK Sheasby A Lumb BM Hunt SP Local Translation in Primary Afferent Fibers Regulates Nociception Plos One 2008 3 e1961 10.1371/journal.pone.0001961 18398477 PMC2276314

[B25] Sutton MA Schuman EM Dendritic protein synthesis, synaptic plasticity, and memory Cell 2006 127 49 58 10.1016/j.cell.2006.09.014 17018276

[B26] Martin KC Casadio A Zhu H Rose JC Chen M Bailey CH Kandel ER Synapse-Specific, Long-Term Facilitation of Aplysia Sensory to Motor Synapses: A Function for Local Protein Synthesis in Memory Storage Cell 1997 91 927 938 10.1016/S0092-8674(00)80484-5 9428516

[B27] Woolf CJ Salter MW Neuroscience - Neuronal plasticity: Increasing the gain in pain Science 2000 288 1765 1768 10.1126/science.288.5472.1765 10846153

[B28] Latremoliere A Woolf CJ Central Sensitization: A Generator of Pain Hypersensitivity by Central Neural Plasticity J pain 2009 10 895 926 10.1016/j.jpain.2009.06.012 19712899 PMC2750819

[B29] Xu JT Zhao XL Yaster M Tao YX Expression and distribution of mTOR, p70S6K, 4E-BP1, and their phosphorylated counterparts in rat dorsal root ganglion and spinal cord dorsal horn Brain Res 2010 1336 46 57 20399760 10.1016/j.brainres.2010.04.010 PMC2874637

[B30] Asante CO Wallace VC Dickenson AH Formalin-induced behavioural hypersensitivity and neuronal hyperexcitability are mediated by rapid protein synthesis at the spinal level Mol Pain 2009 5 27 10.1186/1744-8069-5-27 19500426 PMC2699332

[B31] Xu QH Fitzsimmons B Steinauer J O' Neill A Newton AC Hua XY Yaksh TL Spinal Phosphinositide 3-Kinase-Akt-Mammalian Target of Rapamycin Signaling Cascades in Inflammation-Induced Hyperalgesia J Neurosci 2011 31 2113 2124 10.1523/JNEUROSCI.2139-10.2011 21307248 PMC3097059

[B32] Zimmermann M Ethical Guidelines for Investigations of Experimental Pain in Conscious Animals Pain 1983 16 109 110 10.1016/0304-3959(83)90201-4 6877845

[B33] Ji YH Mansuelle P Terakawa S Kopeyan C Yanaihara N Hsu K Rochat H Two neurotoxins (BMK I and BMK II) from the venom of the scorpion Buthus martensi Karsch: Purification, amino acid sequences and assessment of specific activity Toxicon 1996 34 987 1001 10.1016/0041-0101(96)00065-7 8896191

[B34] Obara I Tochiki KK Geranton SM Carr FB Lumb BM Liu Q Hunt SP Systemic inhibition of the mammalian target of rapamycin (mTOR) pathway reduces neuropathic pain in mice Pain 2011 152 2582 2595 10.1016/j.pain.2011.07.025 21917376

[B35] Mestre C Pelissier T Fialip J Wilcox G Eschalier A A method to perform direct transcutaneous intrathecal injection in rats J Pharmacol Toxicol Methods 1994 32 197 200 10.1016/1056-8719(94)90087-6 7881133

[B36] Chen J Luo C Li HL Chen HS Primary hyperalgesia to mechanical and heat stimuli following subcutaneous bee venom injection into the plantar surface of hindpaw in the conscious rat: a comparative study with the formalin test Pain 1999 83 67 76 10.1016/S0304-3959(99)00075-5 10506673

[B37] Hargreaves K Dubner R Brown F Flores C Joris J A new and sensitive method for measuring thermal nociception in cutaneous hyperalgesia Pain 1988 32 77 88 10.1016/0304-3959(88)90026-7 3340425

[B38] Chung JY Hong SM Choi BY Cho H Yu E Hewitt SM The expression of phospho-AKT, phospho-mTOR, and PTEN in extrahepatic cholangiocarcinoma Clin Cancer Res 2009 15 660 667 10.1158/1078-0432.CCR-08-1084 19147772 PMC7556734

[B39] Brennan GP Jimenez-Mateos EM McKiernan RC Engel T Tzivion G Henshall DC Transgenic overexpression of 14-3-3 zeta protects hippocampus against endoplasmic reticulum stress and status epilepticus in vivo Plos One 2013 8 e54491 10.1371/journal.pone.0054491 23359526 PMC3554740

[B40] Zhang Q Tang X Zhang ZF Velikina R Shi S Le AD Nicotine induces hypoxia-inducible factor-1alpha expression in human lung cancer cells via nicotinic acetylcholine receptor-mediated signaling pathways Clin Cancer Res 2007 13 4686 4694 10.1158/1078-0432.CCR-06-2898 17699846 PMC4166418

[B41] Raghavendra V Tanga RY DeLeo JA Complete Freunds adjuvant-induced peripheral inflammation evokes glial activation and proinflammatory cytokine expression in the CNS Eur J Neurosci 2004 20 467 473 10.1111/j.1460-9568.2004.03514.x 15233755

[B42] Tanga FY Raghavendra V DeLeo JA Quantitative real-time RT-PCR assessment of spinal microglial and astrocytic activation markers in a rat model of neuropathic pain Neurochem Int 2004 45 397 407 10.1016/j.neuint.2003.06.002 15145554

[B43] Jiang F Liu T Cheng M Pang XY Bai ZT Zhou JJ Ji YH Spinal astrocyte and microglial activation contributes to rat pain-related behaviors induced by the venom of scorpion Buthus martensi Karch Eur J Pharmacol 2009 623 52 64 10.1016/j.ejphar.2009.09.028 19782067

[B44] Codeluppi S Svensson CI Hefferan MP Valencia F Silldorff MD Oshiro M Marsala M Pasquale EB The Rheb-mTOR pathway is upregulated in reactive astrocytes of the injured spinal cord J Neurosci 2009 29 1093 1104 10.1523/JNEUROSCI.4103-08.2009 19176818 PMC2682457

[B45] Tezel G Yang X Luo C Cai J Powell DW An astrocyte-specific proteomic approach to inflammatory responses in experimental rat glaucoma Invest Ophthalmol Vis Sci 2012 53 4220 4233 10.1167/iovs.11-9101 22570341 PMC3392010

[B46] Geranton SM Jimenez-Diaz L Torsney C Tochiki KK Stuart SA Leith JL Lumb BM Hunt SP A rapamycin-sensitive signaling pathway is essential for the full expression of persistent pain states J Neurosci 2009 29 15017 15027 10.1523/JNEUROSCI.3451-09.2009 19940197 PMC2830115

[B47] Norsted Gregory E Codeluppi S Gregory JA Steinauer J Svensson CI Mammalian target of rapamycin in spinal cord neurons mediates hypersensitivity induced by peripheral inflammation Neuroscience 2010 169 1392 1402 10.1016/j.neuroscience.2010.05.067 20538043 PMC2914192

[B48] Shin S Wolgamott L Yu Y Blenis J Yoon SO Glycogen synthase kinase (GSK)-3 promotes p70 ribosomal protein S6 kinase (p70S6K) activity and cell proliferation Proc Natl Acad Sci USA 2011 108 E1204 1213 10.1073/pnas.1110195108 22065737 PMC3223461

[B49] Asante CO Wallace VC Dickenson AH Mammalian Target of Rapamycin Signaling in the Spinal Cord Is Required for Neuronal Plasticity and Behavioral Hypersensitivity Associated With Neuropathy in the Rat J Pain 2010 11 1356 1367 10.1016/j.jpain.2010.03.013 20452291 PMC3000494

[B50] Kelleher RJ Govindarajan A Tonegawa S Translational regulatory mechanisms in persistent forms of synaptic plasticity Neuron 2004 44 59 73 10.1016/j.neuron.2004.09.013 15450160

[B51] Kandel ER Neuroscience - The molecular biology of memory storage: A dialogue between genes and synapses Science 2001 294 1030 1038 10.1126/science.1067020 11691980

[B52] Lenz G Avruch J Glutamatergic regulation of the p70S6 kinase in primary mouse neurons J Biol Chem 2005 280 38121 38124 10.1074/jbc.C500363200 16183639

[B53] Lee CC Huang CC Wu MY Hsu KS Insulin stimulates postsynaptic density-95 protein translation via the phosphoinositide 3-kinase-Akt-mammalian target of rapamycin signaling pathway J Biol Chem 2005 280 18543 18550 15755733 10.1074/jbc.M414112200

[B54] Takei N Inamura N Kawamura M Namba H Hara K Yonezawa K Nawa H Brain-derived neurotrophic factor induces mammalian target of rapamycin-dependent local activation of translation machinery and protein synthesis in neuronal dendrites J Neurosci 2004 24 9760 9769 10.1523/JNEUROSCI.1427-04.2004 15525761 PMC6730227

[B55] Tang SJ Reis G Kang H Gingras AC Sonenberg N Schuman EM A rapamycin-sensitive signaling pathway contributes to long-term synaptic plasticity in the hippocampus Proc Natl Acad Sci U S A 2002 99 467 472 10.1073/pnas.012605299 11756682 PMC117583

[B56] Hou LF Klann E Activation of the phosphoinositide 3-kinase-akt-mammalian target of rapamycin signaling pathway is required for metabotropic glutamate receptor-dependent long-term depression J Neurosci 2004 24 6352 6361 10.1523/JNEUROSCI.0995-04.2004 15254091 PMC6729543

[B57] Price TJ Rashid MH Millecamps M Sanoja R Entrena JM Cervero F Decreased nociceptive sensitization in mice lacking the fragile X mental retardation protein: role of mGluR1/5 and mTOR J Neurosci 2007 27 13958 13967 10.1523/JNEUROSCI.4383-07.2007 18094233 PMC2206543

[B58] Liu Y Bai Z-T Feng X-H Li W-P Yang H-T Zhou J-J Ji Y-H Hyper-excitability in low threshold mechanical A fibers is potentially involved in scorpion BmK sting pain Brain Res Bull 2009 80 116 121 10.1016/j.brainresbull.2009.04.007 19393723

