# Supplementary figures and images for: Activation of mammalian target of rapamycin mediates rat pain-related responses induced by BmK I, a sodium channel-specific modulator
Source: Mol Pain. 2013 Oct 8;9:50. doi: 10.1186/1744-8069-9-50 (PMC3842742; doi:10.1186/1744-8069-9-50)

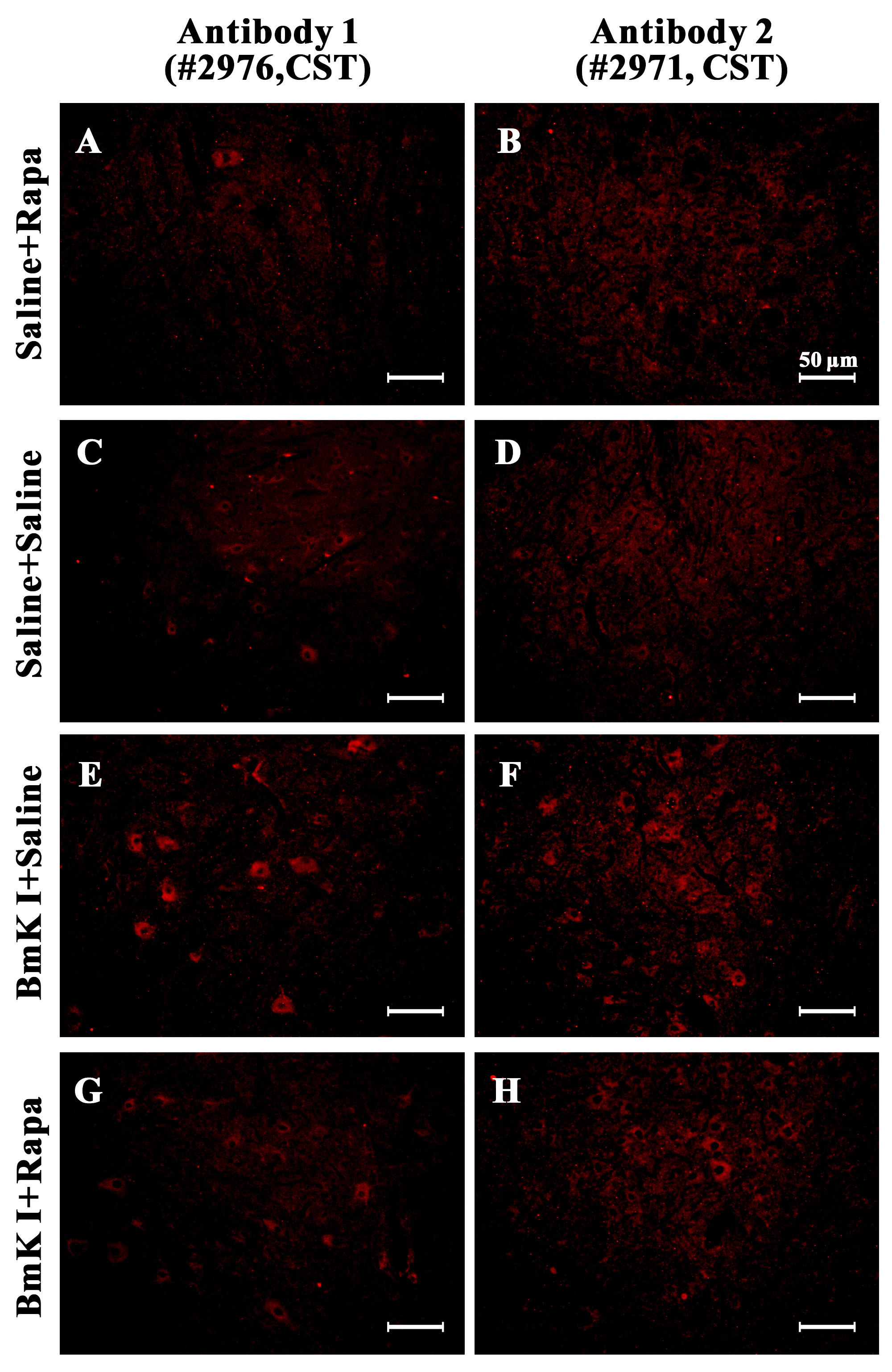

Supplement: Additional file 1: Figure S2 — Immunostaining of p-mTOR was performed by using two individual antibodies from Cell Signaling Technology (CST, antibody 1 catalog #2976, antibody 2 catalog #2971). A&B, animal received i.pl. injection of saline and pre-administration of Rapamycin (i.t.). C&D, animal received i.pl. injection of saline and pre-administration of saline (i.t.). E&F, animal received i.pl. injection of BmK I and pre-administration of saline (i.t.). G&H, animal received i.pl. injection of BmK I and pre-administration of Rapamycin (i.t.). [file 1744-8069-9-50-S1.tiff]

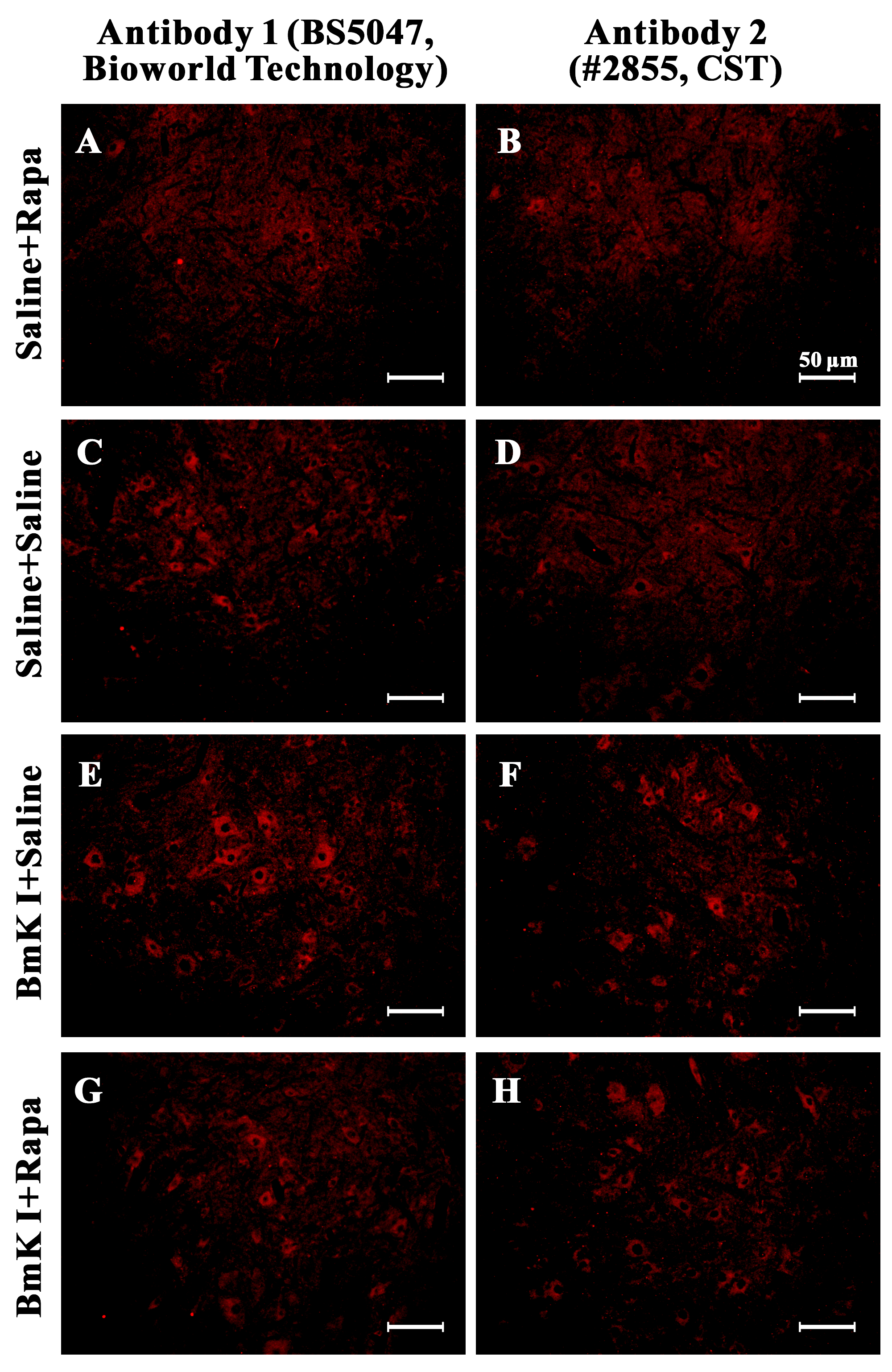

Supplement: Additional file 2: Figure S3 — Immunostaining of p-4E-BP1 was performed by using two individual antibodies: antibody 1 catalog BS5047 from Bioworld Technology and antibody 2 catalog #2855 from CST). A&B, animal received i.pl. injection of saline and pre-administration of Rapamycin (i.t.). C&D, animal received i.pl. injection of saline and pre-administration of saline (i.t.). E&F, animal received i.pl. injection of BmK I and pre-administration of saline (i.t.). G&H, animal received i.pl. injection of BmK I and pre-administration of Rapamycin (i.t.). [file 1744-8069-9-50-S2.tiff]

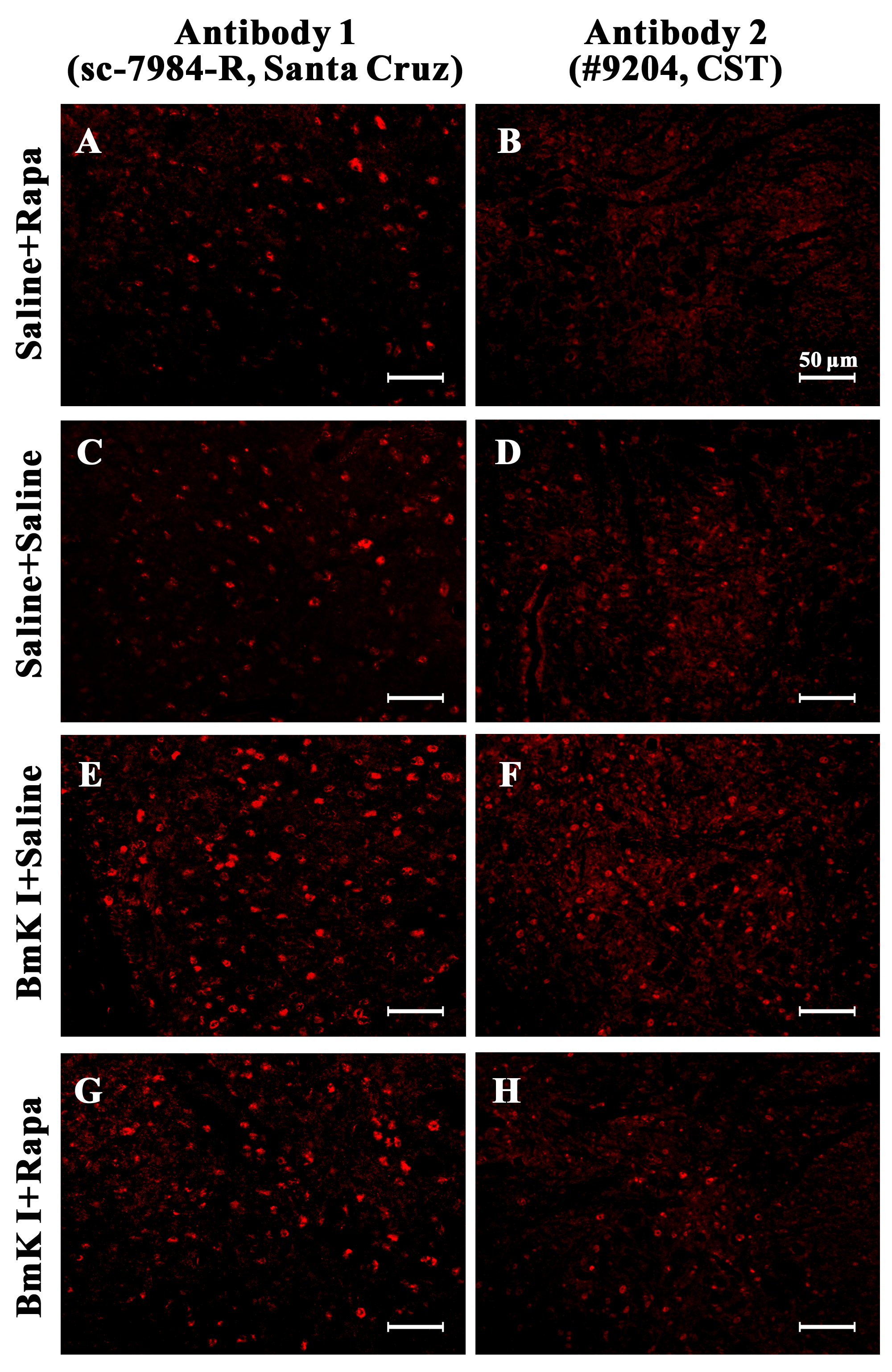

Supplement: Additional file 3: Figure S4 — Activated p70 S6K was stained by two individual antibodies: antibody 1 catalog sc-7984-R from Santa Cruz and antibody 2 catalog #9204 from CST). A&B, animal received i.pl. injection of saline and pre-administration of Rapamycin (i.t.). C&D, animal received i.pl. injection of saline and pre-administration of saline (i.t.). E&F, animal received i.pl. injection of BmK I and pre-administration of saline (i.t.). G&H, animal received i.pl. injection of BmK I and pre-administration of Rapamycin (i.t.). [file 1744-8069-9-50-S3.tiff]

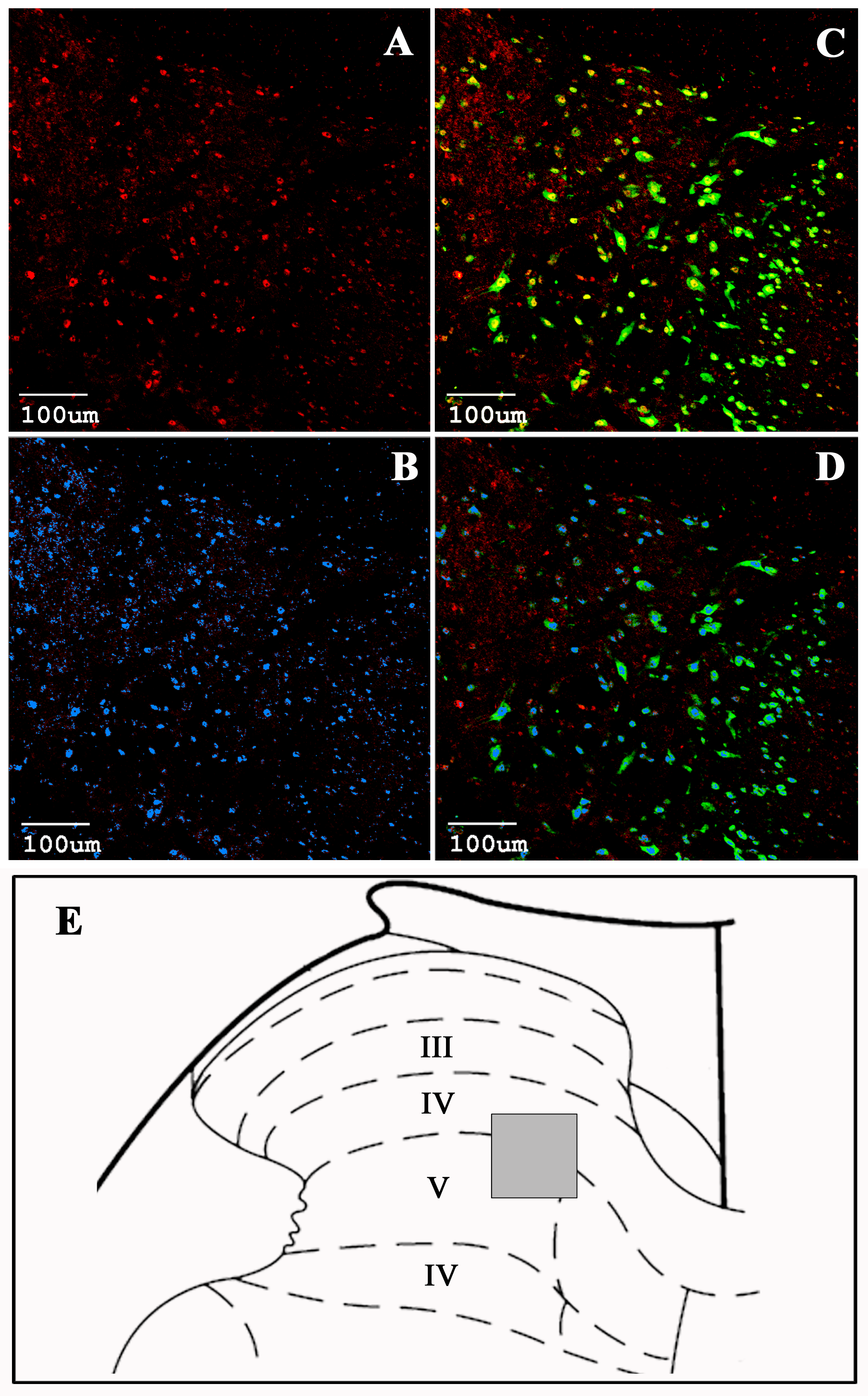

Supplement: Additional file 4: Figure S1 — Illustration of quantitative assessment of double labeling staining. Selected (A) mTOR cascade positive microphotograph and (C) double labeled microphotograph (the same position as shown in A). (B) and (D) Software assisted evaluation of A & B: blue regions were defined as positive signals. (E) The measuring area, as well as the high magnified images in Figure 2, 3 and 4, was captured from lamina III-V of L5 spinal cord dorsal horn. [file 1744-8069-9-50-S4.tiff]
